# Enhanced Efficiency of Anionic Guerbet-Type Amino Acid Surfactants

**DOI:** 10.1021/acs.langmuir.4c02687

**Published:** 2025-01-14

**Authors:** Ettiene Hugo Wiese, Daniel P. Otto, Frans Johannes Smit, Johannes H. L. Jordaan, Hermanus Cornelius M. Vosloo

**Affiliations:** Research Focus Area for Chemical Resource Beneficiation, Catalysis and Synthesis Research Group, North-West University, 11 Hoffman Street, Potchefstroom 2522, South Africa

## Abstract

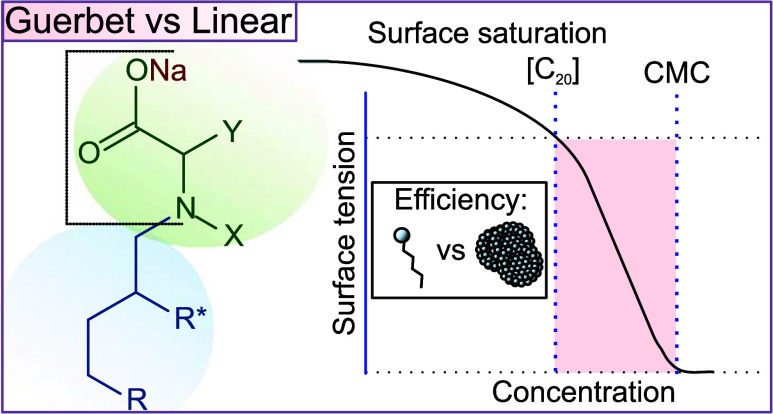

This study investigates the surfactant properties and efficiency of linear and Guerbet-type amino acid surfactants. Utilizing a Wilhelmy plate method, we assessed the colloidal efficiency of these surfactants, with the lowest observed critical micelle concentration at 0.046 mmol L^–1^, significantly reducing surface tension to as low as 25.1 mN·m^–1^. Furthermore, the self-diffusion coefficients of the various surfactants have been determined through ^1^H pulsed-field gradient nuclear magnetic resonance diffusion-ordered spectroscopy. The self-diffusion coefficients are linked to the surface tension reduction as a function of concentration to determine the characteristic time scale of diffusion. In this work, the characteristic time scale of diffusion of a series of surfactants was calculated to investigate the interfacial coverage efficiency. Our findings indicate an inverse relationship between the characteristic time scale of diffusion and critical micelle concentrations across surfactants with hydrocarbon tail lengths of 8–22 carbons. Shorter tails correspond to lower colloidal efficiencies, but rapid surface tension reduction, resulting in the characteristic time scale of diffusion values ranging from 120 ns to 2.15 s. This property is crucial for applications requiring rapid action, such as enhancing aerosol efficiency, improving dispersion, and wetting materials in products.

## Introduction

Today, there is a significant focus on the production of environmentally benign surfactants for their use in for example agricultural products.^1,2^ In the agricultural industry, toxic compounds such as lipophosphides, used as insecticides, are currently being phased out.^3^ Surfactants, especially amino acid surfactants (AASs), could potentially serve as environmentally friendly alternatives in the agricultural sectors such as for aerosol production.^4^ To formulate economically feasible aerosols, it is essential to balance product efficiency and effectiveness, ensuring not only the rapid spreading of the active compound but also its stabilization through methods like emulsification.^5^

The effectiveness of a surfactant in reducing surface tension can be explained by the total reduction in surface tension or surface pressure (Π_CMC_) as determined by tensiometry.^6^ Surfactants that can exert a greater reduction in surface tension on a solvent are considered more effective surfactants. However, the efficiency of surfactants, for example, in stabilizing emulsions or rapidly covering an interface, cannot necessarily be explained from a single perspective. Therefore, a distinction is made between colloidal efficiency (CE) and interfacial coverage efficiency (ICE).

The efficiency of a surfactant varies based on its intended application and can be assessed from multiple perspectives. According to Rosen et al.,^6^ efficiency is determined by the critical micelle concentration (CMC) where a lower CMC indicates higher efficiency. In this article, the efficiency is regarded as CE. According to Ferri and Stebbe,^7^ efficiencies can be influenced by the surfactant monomer concentration and can be determined by the characteristic time scale of diffusion (τ_s_). In this work, the term ICE is introduced to differentiate it from the more commonly used CE, which typically denotes surfactant efficiency. However, CE does not adequately describe the ability of surfactants to quickly form a monolayer covering a surface, hence the need for a new descriptor, ICE.

The CE of a surfactant in forming stable emulsions increases with a decrease in CMC. This means that less of the surfactant is needed to form a stable emulsion. The effectiveness of the surfactants increases with an increase in Π_CMC_. A greater change in surface tension causes the average particle size of the components in the emulsion to decrease, which increases the stability of the emulsion.^8^ However, nanoemulsions are more stable when a highly insoluble dispersed phase, such as silicone oil, is used.^2,9^ Nanoemulsions are resistant to gravitational separation and flocculation, and they exhibit enhanced bioavailability of encapsulated substances.^8^ Thus, they are used in for example the food industry to emulsify insoluble compounds.^8,10,11^ Consequently, for emulsions, a surfactant with the minimum CMC and the maximum Π_CMC_ is sought after.

In contrast to CE, ICE increases with an increase in the CMC relative to the [C_20_].^7^ ICE reflects how fast a surfactant can cover a surface, while [C_20_] represents the concentration required to saturate a surface with surfactants. It is also established that the surface tension of water is reduced by 20 mN·m^–1^ at [C_20_].^6^ The rate at which surfactants cover the surface is influenced by the adsorption depth (*h*). The distance a surfactant must diffuse through a liquid to adsorb at the interface is represented by *h*. An increase in dissolved surfactant monomers in the bulk solution will consequently reduce the average *h*. The surface excess (Γ_M_) also has a certain influence on the number of surfactants in the solution close to the interface. A smaller slope in the surface tension reduction curve, represented by a smaller Γ_M_, indicates a higher concentration of dissolved surfactant monomers.

Surfactants that can rapidly cover an interface are required to enhance the aerosol efficiency of a product.^3,7^ When a liquid is sprayed, the average size of a droplet is rapidly reduced, increasing the surface area of the air–water interface. The size of the interface is limited by the dynamic coverage potential of the surfactants. For anionic surfactants, the rate at which the interface is covered is determined by surfactant diffusion, steric, and electrostatic repulsion forces.

In recent years, ionic liquid surfactants have also gained widespread attention due to their superior properties in various applications compared to conventional surfactants.^12,13^ Though this study focuses on anionic surfactants, their use in ionic liquid surfactants should be investigated in future studies. The parameters studied in this work may contribute to understanding the advanced surface behavior of ionic liquid surfactants and enable more precise control over their physicochemical properties.^13^

In this work, the effectiveness and efficiencies of novel Guerbet-type iminodiacetate and aspartate surfactants are studied and compared to their linear counterparts to investigate the significance of the branched hydrophobic group. To calculate the τ_s_ of the surfactants studied in this work, only anionic surfactants have been investigated, and an approach assuming Fickian diffusion has been taken. This allows for the examination of the significant role that the Guerbet-type hydrophobic group plays in the CMC and τ_s_ of the surfactant. Sodium dodecyl sulfate (SDS) is used as a control as it contains 12 carbons in its linear hydrophobic portion and is therefore easily compared to the Guerbet-type surfactants containing 12 carbons. Unlike most studies that focus primarily on the importance of the CMC, this work demonstrates how significantly the monomer concentration affects the rapid spreading of surfactants. This study also emphasizes the key role that the branched structure of Guerbet-type surfactants plays in improving surfactant efficiency.

## Experimental Section

### Material

The solvents and other chemicals were used without purification or drying unless otherwise specified. Methanol, ethanol, and dichloromethane were obtained from Rochelle Chemicals (Johannesburg, South Africa). Tetrahydrofuran (THF) was dried using a ketal distillation with sodium and benzophenone. Methanol was dried and stored with 5 Å molecular sieves. All starting materials, unless indicated otherwise, were purchased from Sigma-Aldrich (Johannesburg, South Africa). Aldehydes had a food-grade purity of ≥95%, while amino acids were ≥98% pure.

### Synthesis of Surfactants

**Scheme 1:**
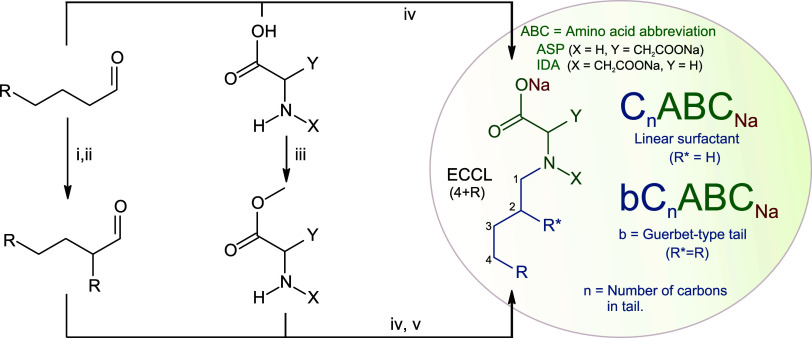
General Reaction Scheme for the Synthesis of AASs^14^ (i) aldol condensation,15 NaOH-EtOH-H_2_O, (ii) hydrogenation,16 H_2_ and Pd–C, (iii) esterification,17 SOCl_2_ and MeOH, (iv) reductive amination,18 α-picoline borane19 and MeOH, (v) saponification, NaOH-EtOH-DCM. (ECCL= Effective carbon chain length).

A general reaction scheme showing the synthesis routes of the AASs investigated in this study is illustrated in Scheme 1. The synthesis of the starting materials and products is beyond the scope of this article, however, detailed information can be found in a publication by Wiese et al.^14−15,16,17,18,19^ Eight AASs were synthesized and compared with SDS, which was purchased from Sigma-Aldrich. All the surfactants were washed with an alkaline ethanol solution to isolate them as sodium salts. The anionic surfactants were then heated to boil in a 10:1 ethanol–water mixture, allowed the mixture to partially evaporate, and then cooled to promote recrystallization. To further improve yield, the remaining surfactant was precipitated by adding acetone to the concentrated ethanol–water solution. Three linear iminodiacetate surfactants (C_8_IDA_Na_, C_11_IDA_Na_, and C_13_IDA_Na_) and four Guerbet-type iminodiacetate surfactants were synthesized (bC_12_IDA_Na_, bC_18_IDA_Na_, bC_20_IDA_Na_, bC_22_IDA_Na_) to investigate the effect of the effective carbon chain length (ECCL) and branching. Furthermore, a Guerbet-type aspartate surfactant (bC_12_ASP_Na_), an isomer of bC_12_IDA_Na_, was synthesized to determine the effect of headgroup symmetry.

### Characterization

Surface tension of the different surfactants was measured at various concentrations using a KRÜSS K100 force tensiometer via the Wilhelmy plate method and calibrated with deionized water at 298.15 K. Before testing, the sample temperature was held steady at 298.15 K with a temperature resolution of 0.01 K and an accuracy of ±0.5 K to allow equilibrium after each dilution. Measurements were taken five times, with a standard deviation of 0.1 mN·m^–1^, and plotted against concentration in mmol L^–1^ on a logarithmic concentration axis. For clarity, concentration values were presented linearly, despite the logarithmic scale.

The self-diffusion coefficients (*D*_0_) for each surfactant were measured using a Bruker Avance NEO 600 MHz nuclear magnetic resonance (NMR) spectrometer. A variety of concentrations were selected to initially determine the self-diffusion coefficients of the monomeric surfactants and to validate the CMCs obtained from tensiometry. Up to 20 different concentrations per surfactant were examined in diffusometry experiments to improve the accuracy of the CMC determinations. Samples, prepared in 5 mm NMR tubes with 0.65 mL of D_2_O, were investigated with an echo gradient pulse program to determine *D*_0_. Proton spectra were recorded at 600 MHz. A broad band direct detection probe, equipped with a *Z*-gradient irradiation coil with a maximum gradient strength (*Z*_m_) of 45 G.cm^–1^, was used to analyze samples. Sixteen different *Z*-gradients were applied between 5–95% relative to *Z*_m_. The gradients were calibrated to the *D*_0_ of the ^1^H signal of the H_2_O impurity in D_2_O (*D*_0_ = 2.29 × 10^–9^ m^2^.s^–1^).^20^ In this experiment, a pulse duration, δ, of 1 ms and a diffusion delay, Δ, of 60 ms were employed. A relaxation delay (d1) of 5 times the longest *T*_1_ relaxation time was allowed between each scan. The DOSY spectra were processed with the Dynamics Center software (Bruker Biospin, Fällanden, Switzerland) where the difference in signal intensity was plotted as a function of surfactant concentration and fitted to the Stejskal-Tanner equation to calculate *D*_0_, eq 1(21)
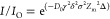
1In the equation, *I* represents the signal intensity at *Z*_m_, and *I*_O_ represents the signal intensity at a zero-gradient strength. The gradient pulse shape (σ) and the gyromagnetic ratio (γ) are constants used to determine *D*_0_ in the above-mentioned equation.

### Calculation of Physicochemical Properties

Since the analysis is conducted in water with anionic surfactants, the Gibbs adsorption isotherm was used to calculate various physicochemical properties.^6^ A simplified approach was taken to calculate the surface excess (Γ_M_) for all anionic surfactants using eq 2, facilitating comparisons of surfactant efficiency^22^
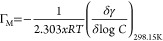
2

In the equation, the change in surface tension (δγ) according to the change in concentration (*δC*) is determined experimentally. *R* and *T* denote the ideal gas constant and temperature, respectively. The parameter *x* represents the number of surface-active species at the interface and is therefore set as *x* = 2 for anionic surfactants and their corresponding cationic counterion. The area per surfactant at the interface (*A*_M_) is calculated using eq 3(23)
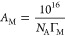
3

The τ_s_ is calculated using eq 4(7)
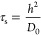
4

To calculate τ_s_, the *D*_0_ of the surfactants in water is experimentally determined. The adsorption depth (*h*) is calculated from a series of equations provided in the Supporting Information.

## Results and Discussion

The comparison of the physicochemical properties of the investigated surfactants was based on the effective carbon chain length (ECCL) and the total number of carbons (*C*_n_) in the hydrophobic portion, or the tail group, of the surfactants. The ECCL is the number of carbons in the longest hydrocarbon chain of the hydrophobic portion of the surfactant and can be calculated using eq 5. In this equation, 4 represents the first 4 carbons in the backbone of the hydrophobic group and *R* represents the number of remaining carbons in the backbone, as illustrated in Scheme 1
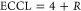
5

*C*_n_ is calculated using eq 6. When calculating *C*_n_ for Guerbet-type surfactants, R* = *R*; however, for linear surfactants, R* = *H*
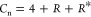
6

**Figure 1:**
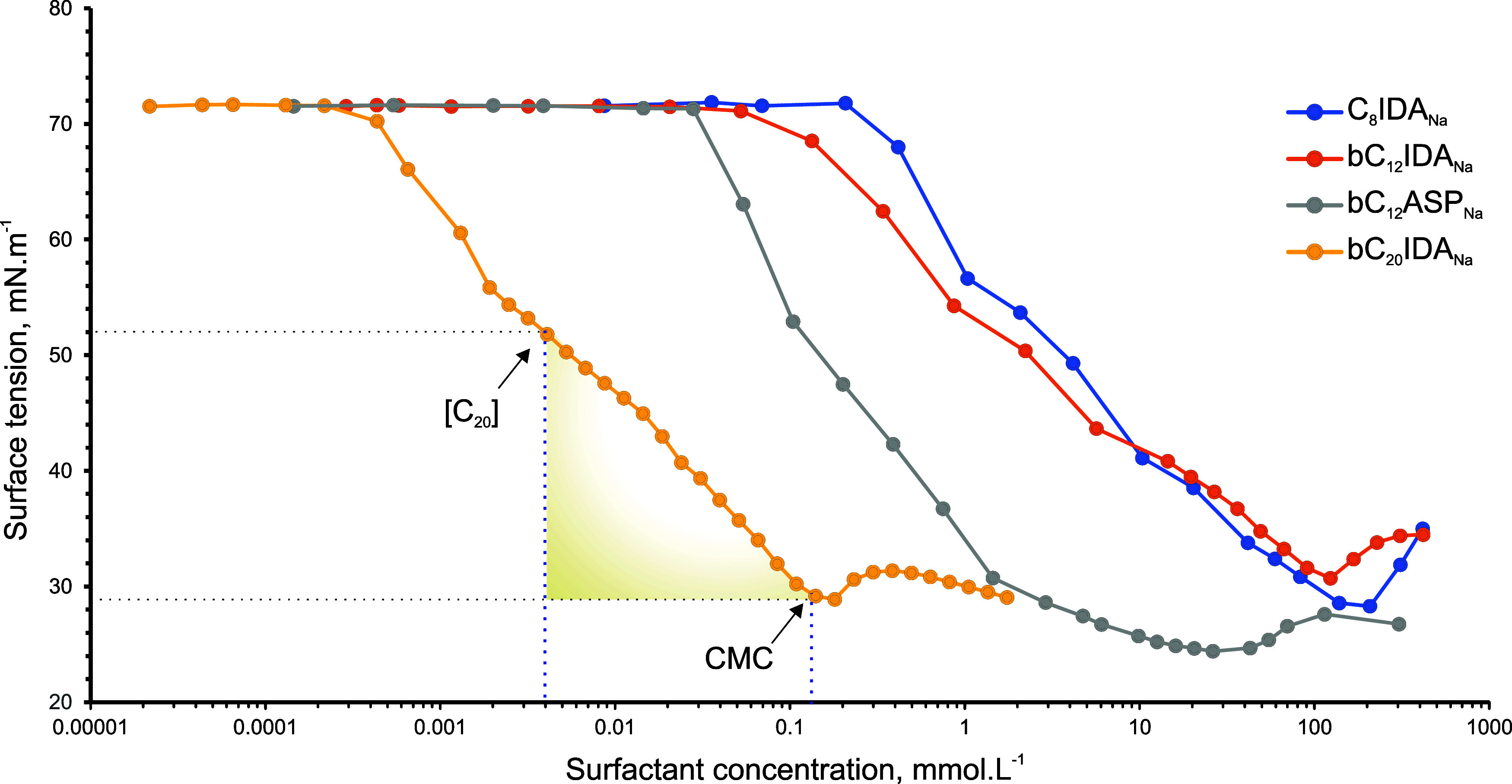
Concentration-dependent surface tension curves of AASs.

From the surface tension data, illustrated in Figure 1, several physicochemical properties can be determined, for example, the CMC, [C_20_], and the slope of surface tension reduction. The study focused on the relationship between the structural characteristics and the physicochemical properties of the surfactants. Initially, it explored the surfactant’s hydrophobicity, particularly examining the carbon chain length and the presence of any branching within the tail. It then compared iminodiacetic acid with aspartic acid to assess the impact of stereochemistry on surfactant efficiency and effectiveness. Finally, the study investigated the influence of surfactant structure on both CE and ICE, aiming to pinpoint a balance or “sweet spot” that optimizes both aspects of surfactant performance.

Typically, the CMC of surfactants is calculated at the inflection point, as shown in Figure 1. For a sodium salt of an amino acid surfactant, a parabola-shaped curve is observed at the CMC. The CMC is identified once the surface tension deviates from the slope established after reaching [C_20_]. If the surface tension does not decrease as expected, it indicates that some surfactants have aggregated. Just before reaching the CMC, the maximum monomer concentration is established in the solution. However, after reaching the CMC, the maximum monomer concentration decreases, causing a slight increase in surface tension as aggregation becomes more favorable. Any additional surfactants added to the solution becomes part of the aggregates.

It was also found that the shape of the parabola significantly differs with a change in the headgroup (bC_12_IDA_Na_ compared to bC_12_ASP_Na_). The difference in the shape of the parabola can be attributed to the distribution of surfactants between the water phase, aggregates, and the air–water interface. After reaching the CMC, the surface tension can moderately decrease, as in the case of the analysis of bC_12_ASP_Na_, since the monomer concentration in the bulk solution can be increased.^6^ The increase in the concentration of sodium cations can reduce the electrostatic repulsion forces between the anionic surfactant at the interface.^24^ Consequently, Γ_M_ can increase after reaching the CMC.

Comparing the ECCL, *C*_n_, or even the presence of branching in the tail of the surfactants with each other, no significant change in the shape of the parabola or the slope of the surface tension reduction are observed. However, not only the CMC but also the concentration at which the surface tension initially decreases is significantly increased with a decrease in ECCL or *C*_n_. It is also important to note that a change in the headgroup affects the slope of the surface tension-concentration curves (bC_12_IDA_Na_ compared to bC_12_ASP_Na_). Therefore, the headgroup has a greater impact on Γ_M_ compared to the tail group. The curves of all the surfactants are provided in the Supporting Information.

**Figure 2:**
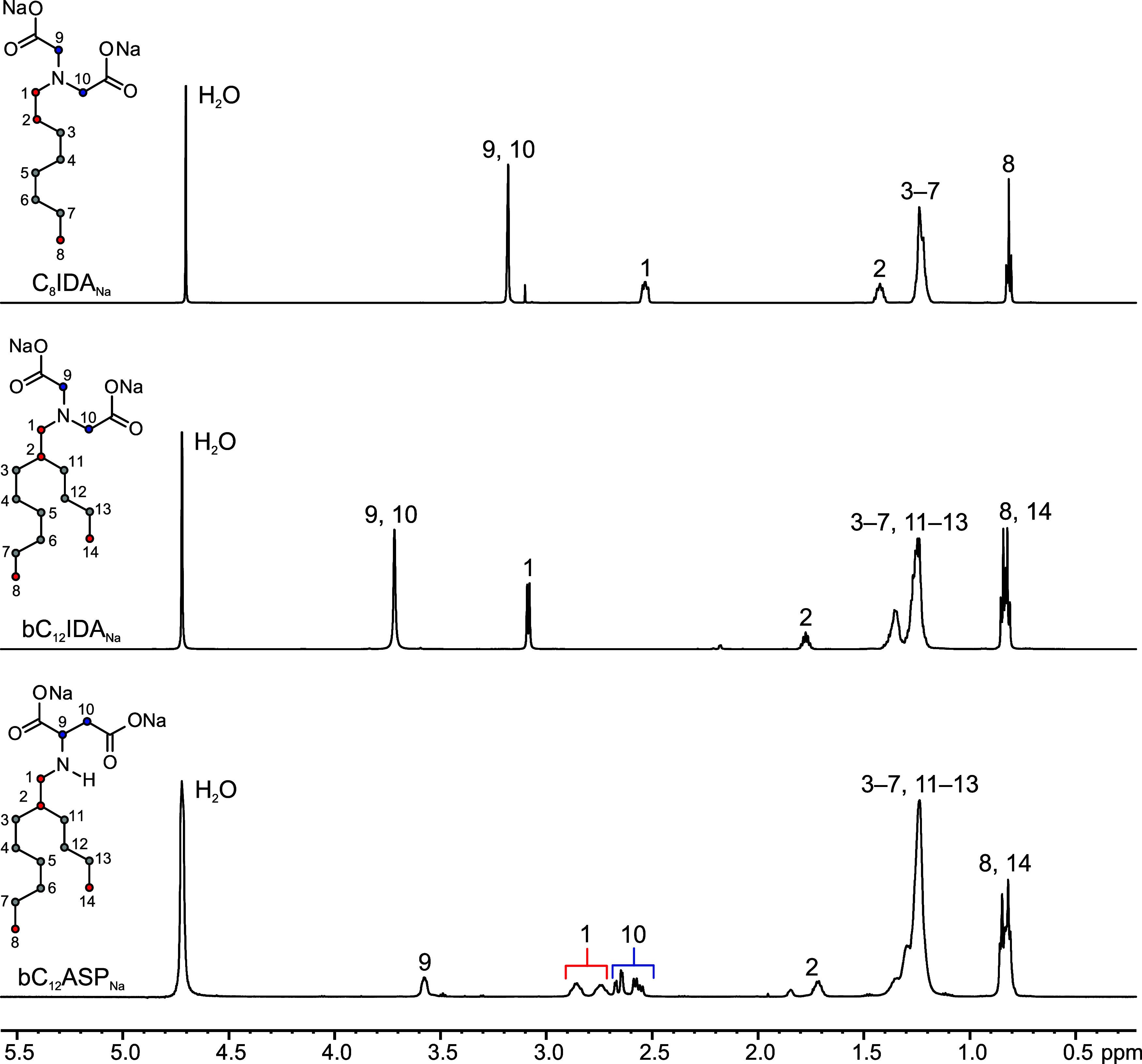
^1^H NMR spectra and structural elucidation of C_8_IDA_Na_, bC_12_IDA_Na_ and bC_12_ASP_Na_ (600 MHz, D_2_O).

While it is commonly assumed that minima in surface tension measurements are indicative of impurities, this interpretation is not universally applicable. More recent studies demonstrated that minima could also arise from factors such as pH of the surfactant solution and the consequent solubility of the ionic surfactant monomers.^25^ The purity of the surfactants was confirmed using ^1^H NMR via a relative integration method, as demonstrated by Leonard et al.^26^ and validated with LC-MS as illustrated in Figures S4.1–S4.12. Representative ^1^H NMR spectra are illustrated in Figure 2, which demonstrates the structural elucidation and purity of the surfactants. The purity of three linear iminodiacetate surfactants, C_8_IDA_Na_ (99%), C_11_IDA_Na_, (99%), C_13_IDA_Na_ (97%) and five Guerbet-type surfactants bC_12_IDA_Na_ (98%), bC_18_IDA_Na_ (94%), bC_20_IDA_Na_ (94%), bC_22_IDA_Na_ (98%), bC_12_ASP_Na_ (94%) were determined. Any impurities detected can be attributed to residual solvents, such as ethanol.

**Figure 3:**
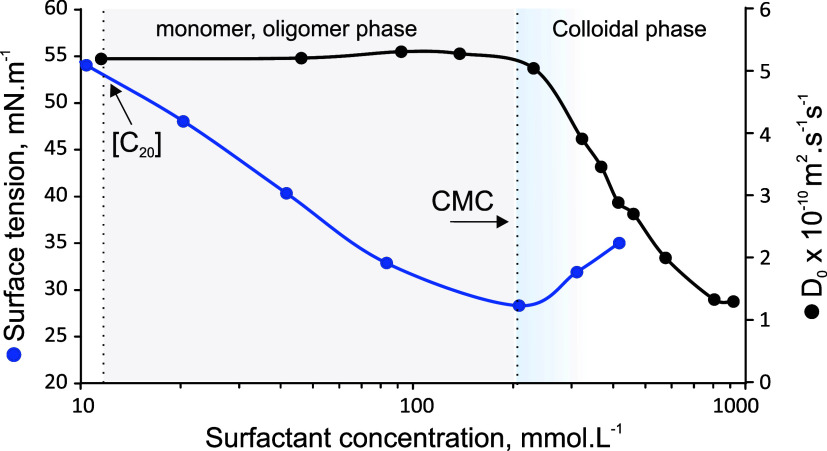
Overlay of diffusometry and tensiometry curves for C_8_IDA_Na_, shown as a representative example.

Diffusometry was used to validate the CMC values determined by tensiometry. Figure 3 shows an overlay of the tensiometric and diffusometric curves for C_8_IDA_Na_, demonstrating that the CMC from tensiometry align well with those from diffusometry. The CMC values for all surfactants, as obtained via diffusometry and detailed in the Supporting Information (Table S3), are of the same order of magnitude as their tensiometric counterparts, with only minor variations. However, tensiometry offers additional valuable insights within the monomeric to oligomeric concentration range, unlike techniques such as diffusometry and conductometry. Therefore, within the context of this research, tensiometry will be used not only to study the CMC but also to examine the [C_20_] value and the reduction in surface tension between the CMC and [C_20_].

### Colloidal Efficiency and Effectiveness of Anionic Surfactants

**Figure 4:**
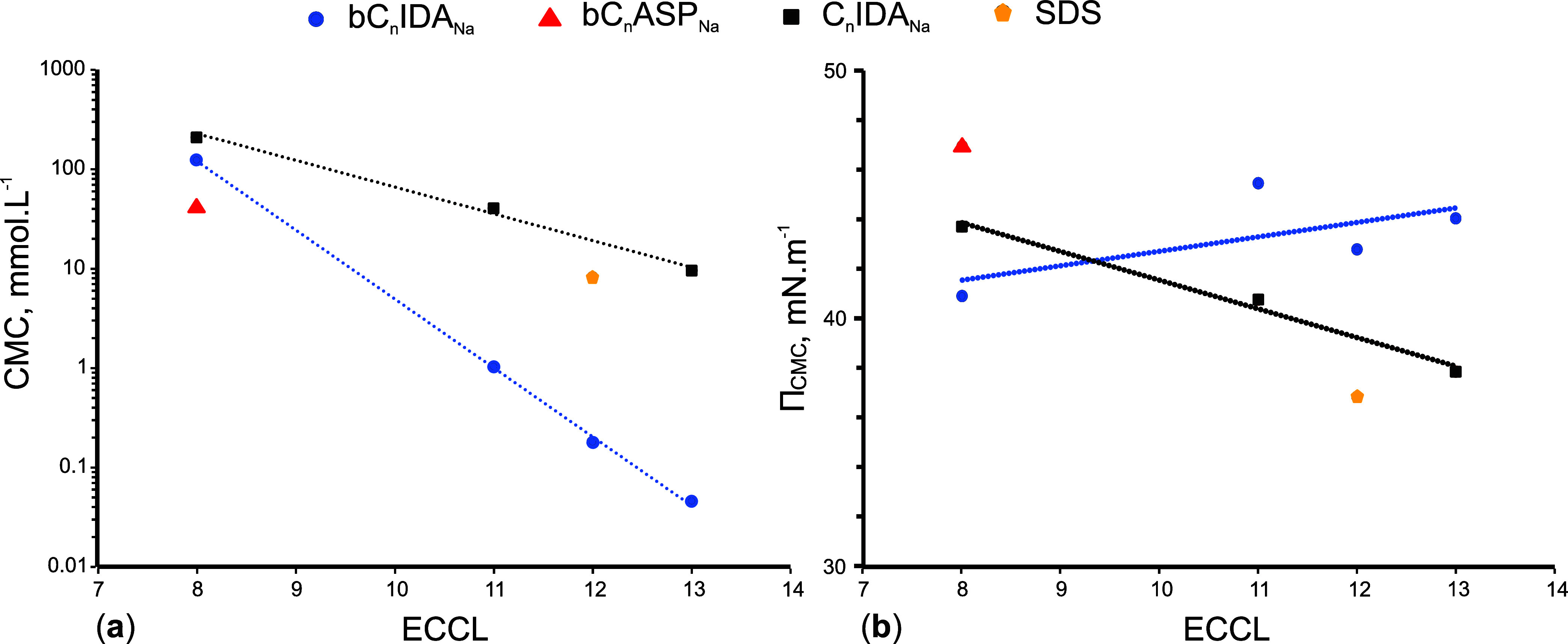
CMC (a) and Π_CMC_ (b) as a function of ECCL for the studied anionic surfactants.

The CE and effectiveness of surfactants are closely tied to their CMC, which can be determined from concentration-dependent curves. The logarithmic relationship between the CMC and the ECCL is depicted in Figure 4a. As mentioned earlier, for the sake of clarity, concentration values are presented in a linear format despite the logarithmic scale of the axis.

The effectiveness of the surfactants can be determined by comparing their Π_CMC_ values with varying ECCL as illustrated in Figure 4b. The surfactants are also compared to SDS, a well-known industrial surfactant. SDS is chosen as a reference surfactant as it is anionic and viewed as an efficient surfactant.^27,28^ The measured CMC of SDS, 8.2 mmol L^–1^, is in good agreement with the values reported in the literature.^27,29^ From Figure 4b, the observation is made that the effectiveness of the surfactants does not show significant differences. However, the effectiveness of bC_12_ASP_Na_, in terms of Π_CMC_, is almost 30% higher than that of SDS. The higher effectiveness can be attributed to a denser packing of the chiral aspartate at the interface or due to an increased monomer solubility.

The tail group of the surfactant, on the other hand, has a remarkable influence on the CE of the surfactants and will be discussed next. As illustrated in Figure 4a, the CMCs of the surfactants with an ECCL of 8 (C_8_IDA_Na_, bC_12_IDA_Na_, and bC_12_ASP_Na_) are relatively similar. However, the Guerbet-type bC_12_IDA_Na_ and bC_12_ASP_Na_ do have lower and consequently more effective CMCs compared to the linear C_8_IDA_Na_. Lower CMCs are, from a certain perspective, more efficient, economical, and environmentally friendly because colloidal properties are achieved at lower concentrations. The reduction in CMC can be attributed to the increased hydrophobicity of the tail group induced by the additional methylene groups present in the branched chain. The CMC is reduced due to an increase in the free energy of the solution. The free energy of the solution increases with an increase in the amount of hydrogen bonds broken between water molecules. These hydrogen bonds are broken by the methylene groups that act as hindrances between the water molecules.^5,6,30^

**Figure 5:**
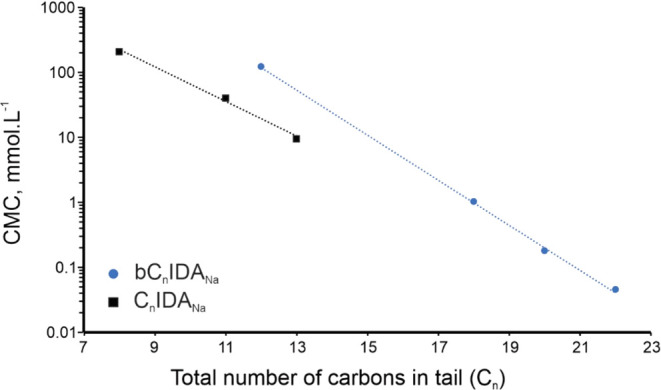
Relationship between the CMC and *C*_n_ of Guerbet-type and linear AASs.

As the ECCL increases, the contrast in the CMC reduction between linear and Guerbet-type surfactants becomes more pronounced. Specifically, for IDA surfactants with an ECCL of 13, the CMC of bC_22_IDA_Na_ is about 200 times lower than its linear counterpart (C_13_IDA_Na_). On the other hand, *C*_n_ can be used as a measure to compare the efficiencies with each other. Figure 5 highlights that linear surfactants, particularly those with lower *C*_n_ values, exhibit substantially lower CMCs compared to their Guerbet-type counterparts. This difference is attributed to the branched tail of the Guerbet-type surfactants, which introduces steric hindrance that impedes aggregation.^31^ However, as the C_n_ values increase, the impact of this branching weakens due to a decreasing ratio of methine branches to methylene groups.^32^ This observation also supports the establishment of a logarithmic relationship between *C*_n_ values and the CMC.

Rosen and Kunjappu^6^ highlighted the predictive capability of *C*_n_ to calculate the CMC of surfactants with similar headgroups, noting a logarithmic correlation between the length of the carbon tail and the CMC.^6^ For the linear surfactant C_22_IDA_Na_, a CMC of 0.042 mmol L^–1^ is anticipated based on a graphical analysis of Figure 5. The CMC for the Guerbet-type surfactant bC_22_IDA_Na_ is measured at 0.046 mmol L^–1^. Consequently, the difference in CMC for surfactants with larger carbon numbers (e.g., C_22_IDA_Na_ versus bC_22_IDA_Na_) is minimal. Although surfactants with lower CMCs are preferred for their emulsifying capabilities, the increased viscosity associated with higher ECCL surfactants may restrict their applicability, as seen with linear variants. Guerbet-type surfactants, in contrast, achieve comparable colloidal stability with reduced viscosity and lower Krafft points.^31−33^ Furthermore, the influence of viscosity and *D*_0_ on ICE is an important consideration and will be discussed next.

### Interfacial Coverage Efficiency of Anionic Surfactants

**Figure 6:**
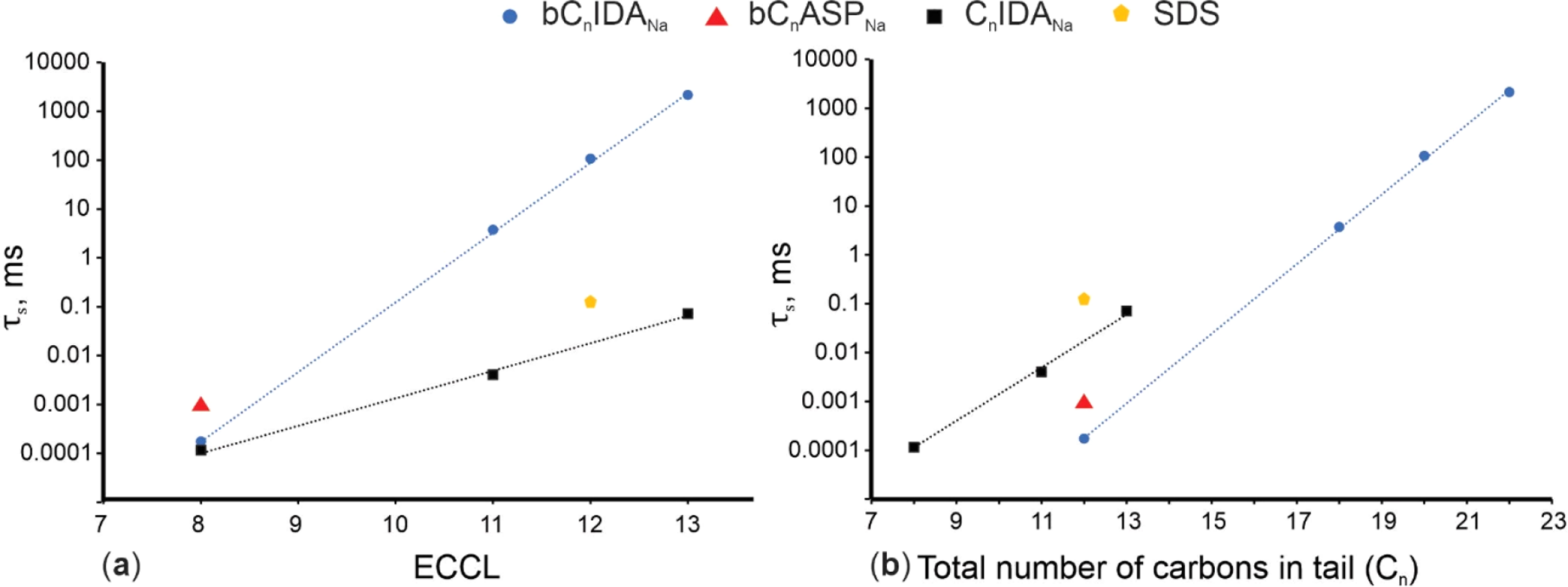
Difference in τ_s_ as a function of ECCL (a) and *C*_n_ (b) for the anionic surfactants.

To facilitate a comparison of the ICE among anionic surfactants, the τ_s_ for each surfactant is calculated. Following the assumption of Fickian diffusion for evaluating a series of negatively charged surfactants, this study has not delved into the influences of various ionic groups or the branching of the surfactant tails on the kinetics of interfacial exchange, as these considerations extend beyond the current scope. Nevertheless, the impact of surfactant hydrophobicity on τ_s_ has been explored. Detailed diffusion time scale curves for all synthesized surfactants can be found in the Supporting Information, with summarized τ_s_ values at the CMC are illustrated in Figure 6.

Figure 6 demonstrates that τ_s_ increases with both the ECCL and *C*_n_. Unlike CE, ICE sees an improvement with a reduction in tail carbon atoms. This enhanced efficiency is linked to a synergy between the monomer’s solubility and the surfactant’s capability to efficiently lower surface tension. There exists an inverse relationship between ECCL (or *C*_n_) and the CMC, indicating that surfactants with higher CMCs contain more dissolved monomers. This results in a reduced average *h*, which in turn, since τ_s_ is related to *h*, means that τ_s_ decreases as CMC increases. The calculated values of *h* are presented in Table 1.

**Table 1:** Physicochemical Properties of the Surfactants

surfactant	ECCL	*C*_n_	(10^–5^ mol L^–1^)	CMC (mmol L^–1^)	*L* (mmol L^–1^)	Γ_m_ (10^–6^ mol.m^–2^)	*h* (μm)	*D*_0_ (10^–10^ m^2^.s^–1^)	τ_s_ (ms)
bC_12_ASP_Na_	8	12	10.4	42.9	42.8	0.91	0.02	4.43	0.001
bC_12_IDA_Na_	8	12	222	123.3	121	1.06	0.009	4.28	0.0002
bC_18_IDA_Na_	11	18	2.27	1.04	1.01	1.29	1.2	4.18	4
bC_20_IDA_Na_	12	20	0.41	0.18	0.18	1.23	6.8	4.32	107
bC_22_IDA_Na_	13	22	0.10	0.05	0.05	1.28	28	3.60	2151
C_8_IDA_Na_	8	8	1039	207.8	197	1.59	0.008	5.03	0.0001
C_11_IDA_Na_	11	11	398	40.3	36.3	1.74	0.04	4.62	0.004
C_13_IDA_Na_	13	13	122	9.5	8.29	1.78	0.19	4.91	0.07
SDS	12	12	189	8.2	6.31	2.23	0.27	5.77	0.1

It is important to note that ICE is also influenced by the structure of the tail group (b*C*_n_IDA_Na_ versus *C*_n_IDA_Na_). The Guerbet-type surfactants consistently have higher ICEs compared to the linear surfactants when using *C*_n_ as a reference point (Figure 6b). Additionally, it was observed that all synthesized surfactants with a *C*_n_ of 12 have better ICE than SDS. Furthermore, linear surfactants exhibit better ICE at an ECCL greater than 7, as shown in Figure 6a. A convergence between the τ_s_ values of Guerbet-type versus linear surfactants, in terms of ECCL, occurs. Therefore, it is postulated that a b*C*_n_IDA_Na_ surfactant with an ECCL equal to or smaller than 7 has better interfacial coverage efficiency compared to *C*_n_IDA_Na_ surfactants, in terms of both ECCL and *C*_n_.

It is important to consider all the calculated parameters illustrated in Table 1 to understand the rate-determining factors of interfacial adsorption. [C_20_] decreases with an increase in ECCL or *C*_n_ of a surfactant. Therefore, one might argue that less surfactant is required to cover the surface, making the surfactants more effective. However, it has been observed that several factors come into play and will now be explained.

After reaching [C_20_], any additional surfactants added to the solution become part of the bulk solution. These surfactant monomers are available for adsorption if for example the interfacial area of the air–water interface increases. Should the concentration rise beyond the CMC, these aggregated surfactants then serve as reservoirs of surfactants available for adsorption. However, the viscosity of the solution increases as the number of aggregates in suspension increases, which hinders rapid adsorption. This study investigates the concentration of surfactant monomers in the bulk solution that are available for interfacial coverage. This concentration, denoted as *L*, can be accurately calculated using eq 7
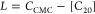
7As the concentration of available monomers in the bulk solution increases, both the average *h* and the τ_*s*_ decrease. This reduction in *h* signifies that surfactants have a shorter diffusion distance to reach the interface, facilitating quicker adsorption. Given that the surfactants generally exhibit similar *D*_0_, the role of *h* becomes crucial in influencing τ_*s*_. An ideal surfactant for achieving optimal ICE would therefore maintain a minimal [C_20_] ratio relative to the concentration of available monomers in the solution (*L*).

The role of the headgroup is essential for the solubility of surfactant monomers. The [C_20_] of bC_12_IDA_Na_ and SDS is similar, although the τ_s_ of bC_12_IDA_Na_ is nearly 700 times lower and therefore more efficient than that of SDS. Thus, the available surfactant monomers in the bulk solution of bC_12_IDA_Na_ are almost 20 times higher compared to SDS. The two carboxylate groups in the headgroup of bC_12_IDA_Na_ facilitate high solubility relative to the surfactant’s hydrophobic tail. Therefore, when designing efficient interfacial coverage surfactants, one must take the balance between the hydrophobic and hydrophilic groups into consideration.

## Conclusions

In conclusion, this study investigated a series of unique amino acid surfactants, revealing a wide range of CMCs from 0.05–208 mmol L^–1^. The research confirms that Guerbet-type surfactants demonstrate superior CE when ECCL is considered, and linear surfactants prove more efficient when *C*_n_ is less than 22. Notably, the surfactant bC_22_IDA_Na_ exhibited the highest colloidal efficiency, with a CMC approximately 180 times smaller and therefore more efficient than SDS. Furthermore, the τ_s_ varied significantly across the study, with values ranging from 0.12 μs to 2.15 s. C_8_IDA_Na_ was identified as the surfactant with the highest ICE, exhibiting a τ_s_ approximately 1100 times smaller than that of SDS. This study also highlighted that Guerbet-type surfactants display higher ICE compared to linear surfactants at an ECCL smaller than 8. Additionally, the study underlined the importance of the ratio of *L* relative to [C_20_] in determining *h* and thus enhancing ICE.

The AASs examined in this study demonstrate strong potential as efficient alternatives to conventional surfactants, due to their tailored performance in specific applications. The increase in *L*, achieved by increasing the solubility through more ionizable groups in the head or branching in the tail, significantly impacts the surfactant’s performance. This study also suggests that CE and ICE are somewhat inversely related, highlighting that the efficiency of surfactants can vary based on the intended application. Future research should continue to explore the structural influences on surfactant efficiency and extend investigations into the effects of ionic groups and tail branching to fully optimize the use of AASs in various industries

## Supporting Information Available

Surface tension versus concentration curves; self-diffusion versus concentration curves and characteristic time scale of diffusion curves for the linear and Guerbet-type amino acid surfactants; extended formulas for calculations; method and equipment information for DOSY and tensiometry analysis; purity and structural characterization via ^1^H NMR and LC-MS (PDF)

## References

[ref1] Rhein L. D.; Schlossman M.; O’Lenick A.; Somasundaran P. In Surfactants in Personal Care Products and Decorative Cosmetics, 3rd ed.; CRC Press: Boca Raton, 2006; p 504.

[ref2] Gutiérrez J.; González C.; Maestro A.; Solè I.; Pey C.; Nolla J. Nano-emulsions: New applications and optimization of their preparation. Curr. Opin. Colloid Interface Sci. 2008, 13 (4), 245–251. 10.1016/j.cocis.2008.01.005.

[ref3] Tadros T. F. In Surfactants in Agrochemicals, 1st ed.; Routledge: New York, 1995; p 284.

[ref4] Guo J.; Sun L.; Zhang F.; Sun B.; Xu B.; Zhou Y. Progress in synthesis, properties and application of amino acid surfactants. Chem. Phys. Lett. 2022, 794, 139499 10.1016/j.cplett.2022.139499.

[ref5] Myers D. In Surfactant Science and Technology; John Wiley & Sons, 2020.

[ref6] Rosen M. J.; Kunjappu J. T. In Surfactants and Interfacial Phenomena; John Wiley & Sons, 2012.

[ref7] Ferri J. K.; Stebe K. J. Which surfactants reduce surface tension faster? A scaling argument for diffusion-controlled adsorption. Adv. Colloid Interface Sci. 2000, 85, 61–97. 10.1016/S0001-8686(99)00027-5. 10696449

[ref8] de Oca-Ávalos J. M. M.; Candal R. J.; Herrera M. L. Nanoemulsions: stability and physical properties. Curr. Opin. Food Sci. 2017, 16, 1–6. 10.1016/j.cofs.2017.06.003.

[ref9] Lifshitz I. M.; Slyozov V. V. The kinetics of precipitation from supersaturated solid solutions. J. Phys. Chem. Solids 1961, 19 (1–2), 35–50. 10.1016/0022-3697(61)90054-3.

[ref10] Garavand F.; Jalai-Jivan M.; Assadpour E.; Jafari S. M. Encapsulation of phenolic compounds within nano/microemulsion systems: A review. Food Chem. 2021, 364, 130376 10.1016/j.foodchem.2021.130376. 34171813

[ref11] Wiese E.; Venter D.; Otto D.; Smit F.; Vosloo H. Werking van surfaktante in waterige oplossings en by die lug-water-tussenvlak:’n Oorsig. S. Afr. J. Sci. Technol. 2023, 42, 77–87.

[ref12] Buettner C. S.; Cognigni A.; Schröder C.; Bica-Schröder K. Surface-active ionic liquids: A review. J. Mol. Liq. 2022, 347, 118160 10.1016/j.molliq.2021.118160.

[ref13] El Seoud O. A.; Keppeler N.; Malek N. I.; Galgano P. D. Ionic liquid-based surfactants: Recent advances in their syntheses, solution properties, and applications. Polymers 2021, 13 (7), 1100 10.3390/polym13071100. 33808369 PMC8036849

[ref14] Wiese E.; Otto D.; Smit F.; Jordaan J.; Marx F.; Young D.; Vosloo H. Sintese en karakterisering van N-alkielaminosuurderivate met ‘n fokus op Guerbet-tipe variante: Vordering met die vervaardiging van potensiële, volhoubare surfaktante. S. Afr. J. Sci. Technol. 2024, 43, 85–101. 10.36303/SATNT.2024.43.1.974.

[ref15] Foley P.; Yang Y. Guerbet Alcohols and Methods for Preparing and Using Same. U.S. Patent US9840449B2, 2017.

[ref16] Spengler J.; Albericio F. Synthesis of All the Diastereomers of 2-Amino-3-hydroxy-4, 5-dimethylhexanoic Acid. Eur. J. Org. Chem. 2014, 2014, 44–47. 10.1002/ejoc.201301257.

[ref17] Campana Filho S. P.; Goissis G. Kinetics and yield of the esterification of amino acids with thionyl chloride in n-propanol. J. Chromatogr. A 1982, 236, 197–200. 10.1016/S0021-9673(00)82514-5.

[ref18] Sato S.; Sakamoto T.; Miyazawa E.; Kikugawa Y. One-pot reductive amination of aldehydes and ketones with α-picoline-borane in methanol, in water, and in neat conditions. Tetrahedron 2004, 60, 7899–7906. 10.1016/j.tet.2004.06.045.

[ref19] Ramachandran P. V.; Kulkarni A. S. Open-Flask Synthesis of Amine–Boranes via Tandem Amine–Ammonium Salt Equilibration–Metathesis. Inorg. Chem. 2015, 54, 5618–5620. 10.1021/acs.inorgchem.5b00572. 26010677

[ref20] Holz M.; Heil S. R.; Sacco A. Temperature-dependent self-diffusion coefficients of water and six selected molecular liquids for calibration in accurate 1H NMR PFG measurements. Phys. Chem. Chem. Phys. 2000, 2 (20), 4740–4742. 10.1039/b005319h.

[ref21] Sinnaeve D. The Stejskal–Tanner equation generalized for any gradient shape—an overview of most pulse sequences measuring free diffusion. Concepts Magn. Reson., Part A 2012, 40A, 39–65. 10.1002/cmr.a.21223.

[ref22] Schott H. Saturation adsorption at interfaces of surfactant solutions. J. Pharm. Sci. 1980, 69, 852–854. 10.1002/jps.2600690729. 7391954

[ref23] Dahanayake M.; Cohen A. W.; Rosen M. J. Relationship of structure to properties of surfactants. 13. Surface and thermodynamic properties of some oxyethylenated sulfates and sulfonates. J. Phys. Chem. A 1986, 90, 2413–2418. 10.1021/j100402a032.

[ref24] Khan H.; Seddon J. M.; Law R. V.; Brooks N. J.; Robles E.; Cabral J. T.; Ces O. Effect of glycerol with sodium chloride on the Krafft point of sodium dodecyl sulfate using surface tension. J. Colloid Interface Sci. 2019, 538, 75–82. 10.1016/j.jcis.2018.11.021. 30500469

[ref25] Beunen J. A.; Mitchell D. J.; White L. R. Surface tension minimum in ionic surfactant systems. J. Chem. Soc., Faraday Trans. 1978, 74, 2501–2517. 10.1039/f19787402501.

[ref26] Leonard J.; Lygo B.; Procter G. Advanced Practical Organic Chemistry; CRC press, 2013.

[ref27] Cifuentes A.; Bernal J. L.; Diez-Masa J. C. Determination of critical micelle concentration values using capillary electrophoresis instrumentation. Anal. Chem. 1997, 69 (20), 4271–4274. 10.1021/ac970696n.

[ref28] Malik N. A. Surfactant–amino acid and surfactant–surfactant interactions in aqueous medium: a review. Appl. Biochem. Biotechnol. 2015, 176, 2077–2106. 10.1007/s12010-015-1712-1. 26160314

[ref29] Li Y.; Bloor D. M.; Wyn-Jones E. Polymer/surfactant interactions. The controlled desorption of sodium dodecyl sulfate (SDS) from a polymer/SDS complex in aqueous solution. Langmuir 1996, 12 (18), 4476–4478. 10.1021/la960138o.

[ref30] Tadros T. F. Applied Surfactants. Principles and Applications. By Tharwat F. Tadros.. Angew. Chem., Int. Ed. 2006, 5922 10.1002/anie.200585309.

[ref31] O’Lenick A. J. Guerbet chemistry. J. Surfactants Deterg. 2001, 4, 311–315. 10.1007/s11743-001-0185-1.

[ref32] Varadaraj R.; Bock J.; Valint P. Jr; Zushma S.; Thomas R. Fundamental interfacial properties of alkyl-branched sulfate and ethoxy sulfate surfactants derived from Guerbet alcohols. 1. Surface and instantaneous interfacial tensions. J. Phys. Chem. A 1991, 95, 1671–1676. 10.1021/j100157a033.

[ref33] Patil S.; Rajurkar K.; Patil S.; Pratap A. Synthesis of guerbet esters and its application in drilling and grinding oil. Tribol. Int. 2023, 177, 107993 10.1016/j.triboint.2022.107993.

