# Supplementary material for: Enhanced Efficiency of Anionic Guerbet-Type Amino Acid Surfactants
Source: Langmuir. 2025 Jan 14;41(3):1547–55. doi: 10.1021/acs.langmuir.4c02687 (PMC11781027; doi:10.1021/acs.langmuir.4c02687)
Supplement: Supplementary file 1 — la4c02687_si_001.pdf [file la4c02687_si_001.pdf]

Supporting Information

Enhanced Efficiency of Anionic Guerbet-type Amino Acid Surfactants

Ettiene Hugo Wiese, Daniel P. Otto, Frans Johannes Smit*, Johannes Hendrik L. Jordaan and Hermanus Cornelius M. Vosloo*

Research Focus Area for Chemical Resource Beneficiation, Catalysis and Synthesis Research Group, North-West University, 11 Hoffman Street, Potchefstroom, 2522, South Africa

*Email: Daniel.Otto@nwu.ac.za; Tel.: +2718 299 2361 or Frans.Smit@nwu.ac.za

Table of contents:

Method for Tensiometer Analysis……………………………………………………………… S2

Surface tension concentration curves………………………………………………………. S3

Calculation of Physicochemical Properties ………………………………………………… S5

Characteristic timescale of diffusion curves ……………………………………………….. S6

Method for DOSY-analysis and determined CMCs ……………………………………… S8

Self-diffusion concentration curves …………………………………………………………… S9

Method for ^{1}H NMR and LC-MS ………………………………………………………….. S11

^{1}H-NMR characterization ………………………………………………………………… S13

LC-MS spectra ……………………………………………………………………………….. S17

Supporting Information 1: Tensiometry

Wilhelmy plate method

Table S1. Method for Tensiometer Analysis

**Equipment**	
Tensiometer	Kruss K100 Force Tensiometer
Automatic Sampler	Kruss micro dispenser
Temperature Controller	TJ50 Peltier
Probe	Wilhelmy-plate
Solvent	Deionized water
**Settings**	
Temperature	25 °C
Standard deviation	0.1 $mN.m^{-1}$

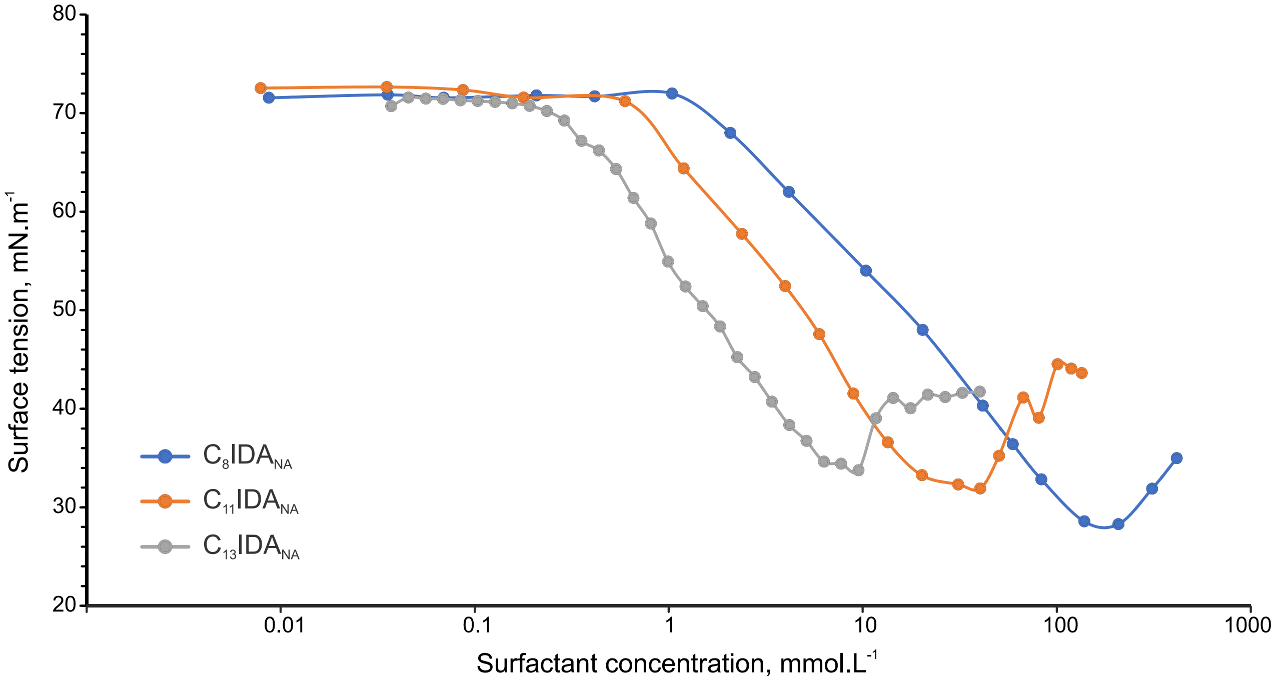

Figure S1.1. Surface tension concentration curves of the linear surfactants.

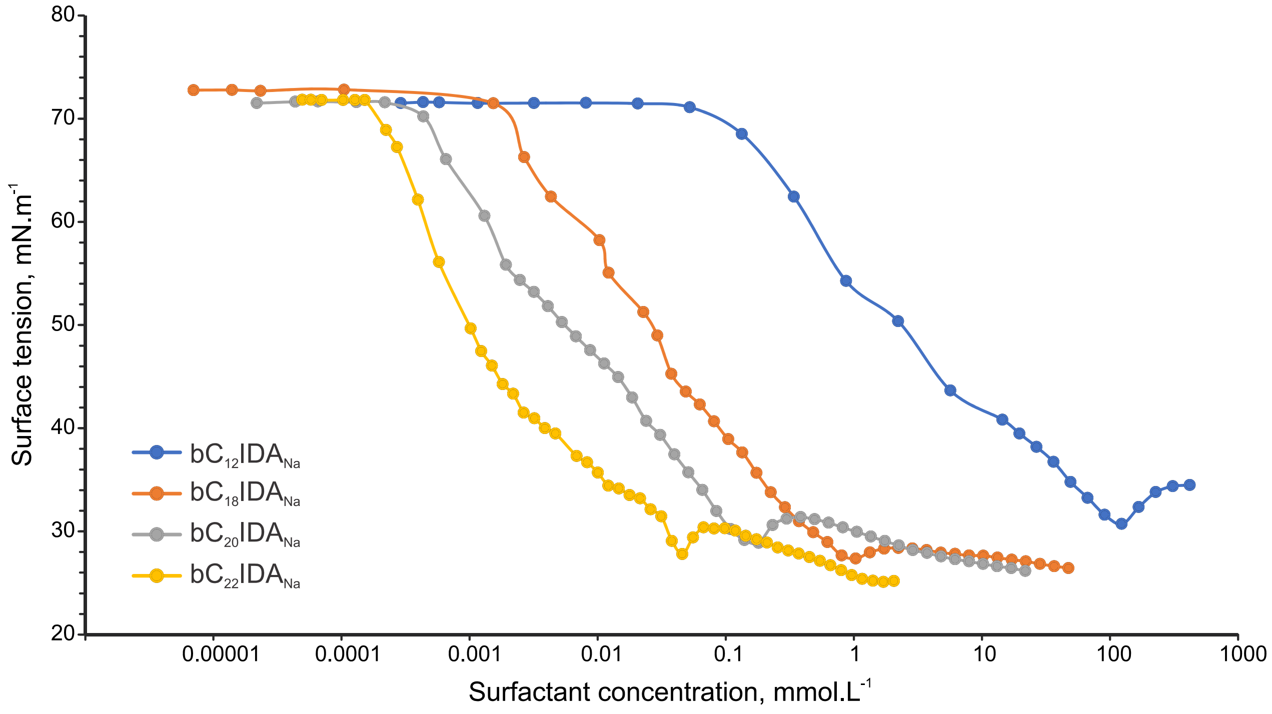

Figure S1.2. Surface tension concentration curves of the Guerbet-type surfactants.

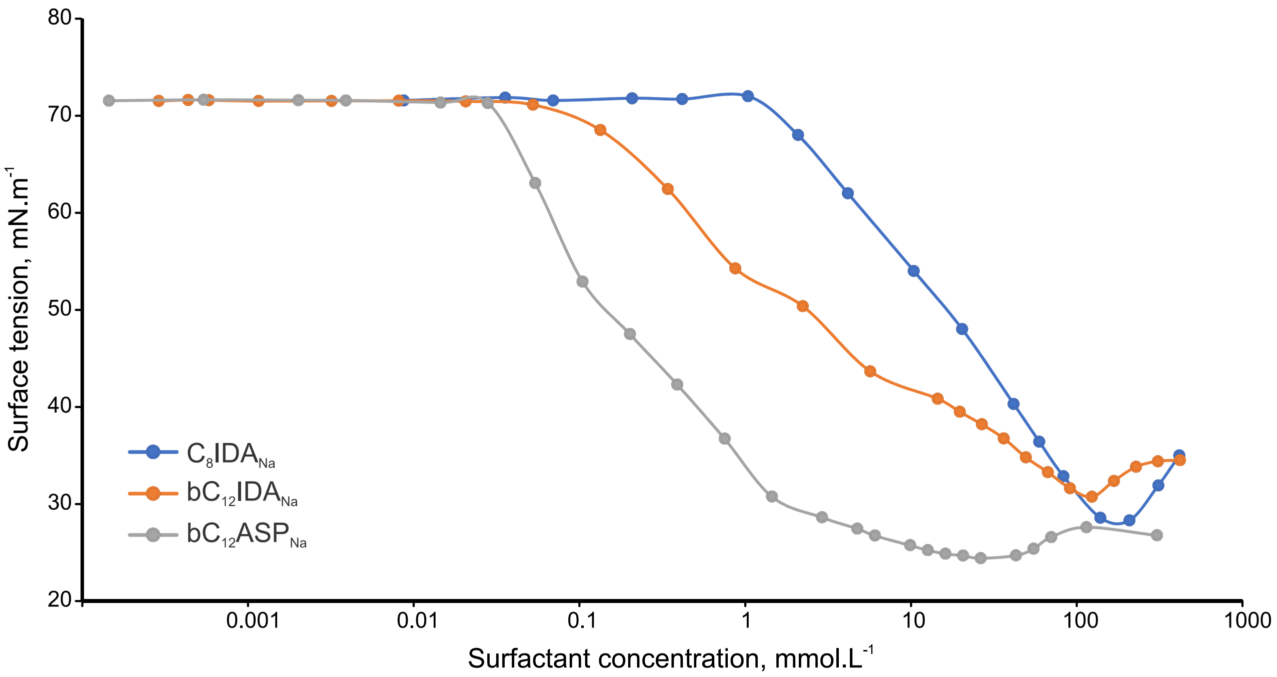

Figure S1.3. Surface tension concentration curves of surfactants with an ECCL of 8.

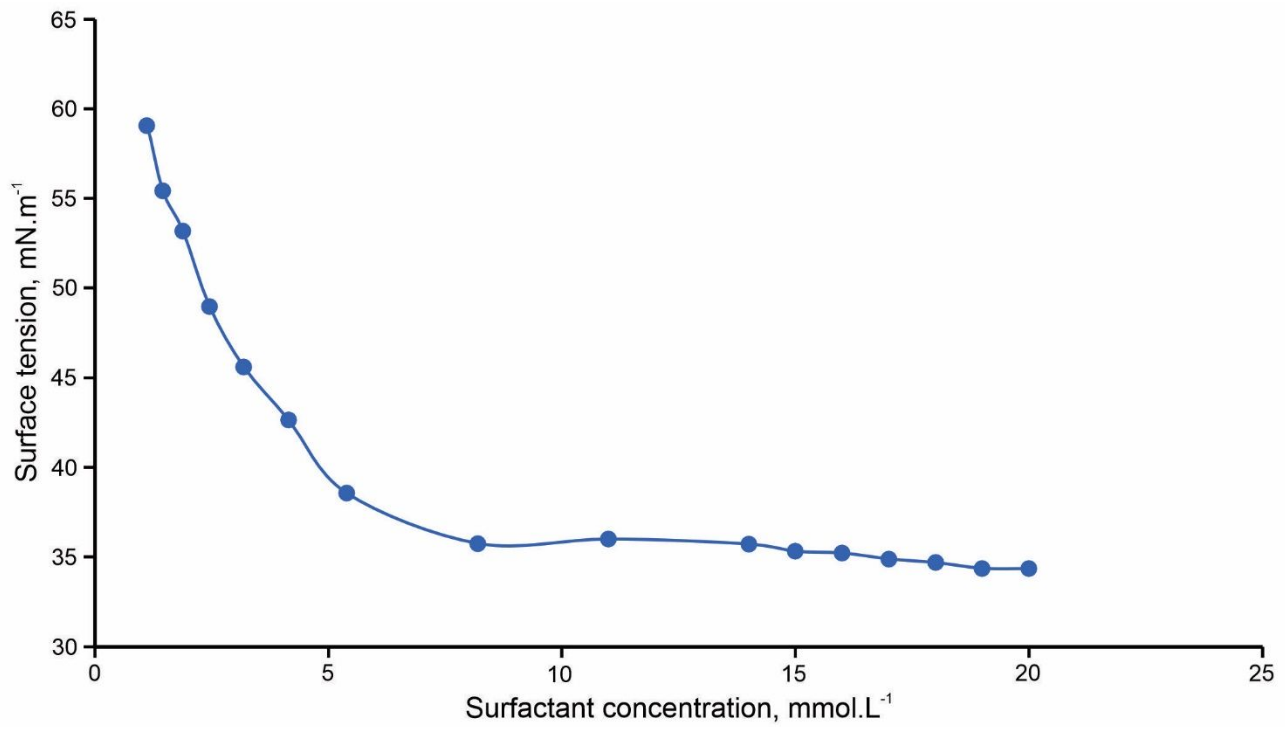

Figure S1.4. Surface tension concentration curve of SDS.

Supporting Information 2: Characteristic time scale of diffusion

Calculation of Physicochemical Properties:

The Gibbs adsorption isotherm was used to calculate the physicochemical properties of the anionic surfactants. The surface excess, Γ_M, was determined using an approximation expressed in eq S1:

$$\Gamma_M = -\frac{1}{2.303\ x\ RT}\left(\frac{\delta\gamma}{\delta logC}\right)_{298.15\ K} \quad \text{(S1)}$$

In the equation, the change in surface tension ($\delta\gamma$) relative to the change in concentration (δC) is experimentally determined using a tensiometer. The parameter x represents the number of surface-active species at the interface and is therefore set as x = 2, for anionic surfactants and their corresponding salts. *R* and *T* represent the ideal gas constant and temperature, respectively. The characteristic timescale for diffusion (τ_s) is calculated using eq S2:

$$\tau_s = \frac{h^2}{D_0} \quad \text{(S2)}$$

To calculate τ_s, the self-diffusion constant (D_0) of the surfactants in water is experimentally determined using NMR. This data is provided in **Supporting Information 3**. The adsorption depth (*h*) is calculated using eq S3:

$$h = \frac{\Gamma_M/a}{1+ C/a} \quad \text{(S3)}$$

The air-water partition coefficient of the surfactant (a) is used to determine h and a is calculated using eq S4:

$$a = \frac{KMK}{10^y - 1} \quad \text{(S4)}$$

To determine a, y is calculated using eq S5:

$$y = \frac{0.072 - \gamma_C}{2.3\ RT\ \Gamma_M} \quad \text{(S5)}$$

To calculate y, the surface tension at C (γ_C) is experimentally determined. Thus, these parameters can be used to determine the τ_s of the surfactants at different concentrations. The calculations are based on the research by Ferri and Stebbe.[1]

1. J. K. Ferri en K. J. Stebe, *Adv. Colloid Interface Sci.,* **2000**, *85* (1), 61-97.

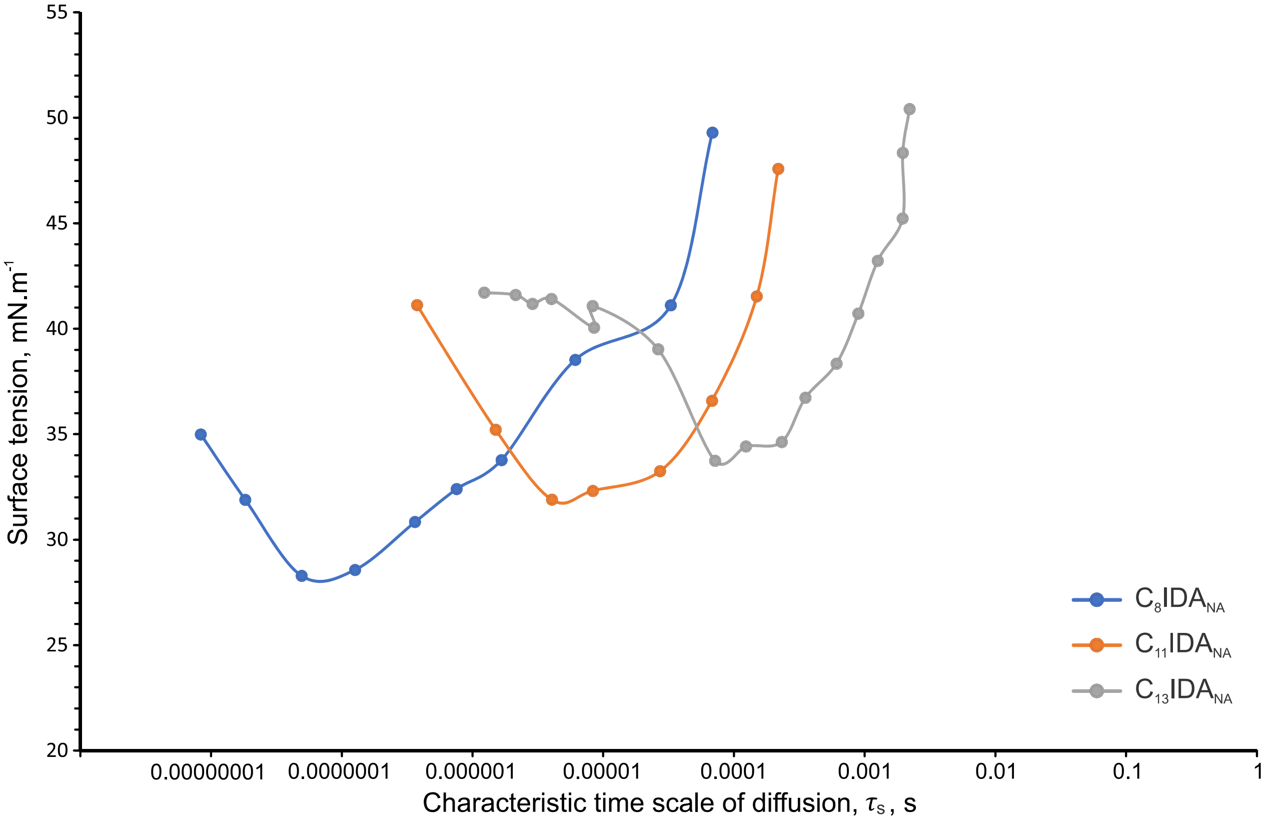

Figure S2.1. Characteristic timescale of diffusion curves of the linear surfactants.

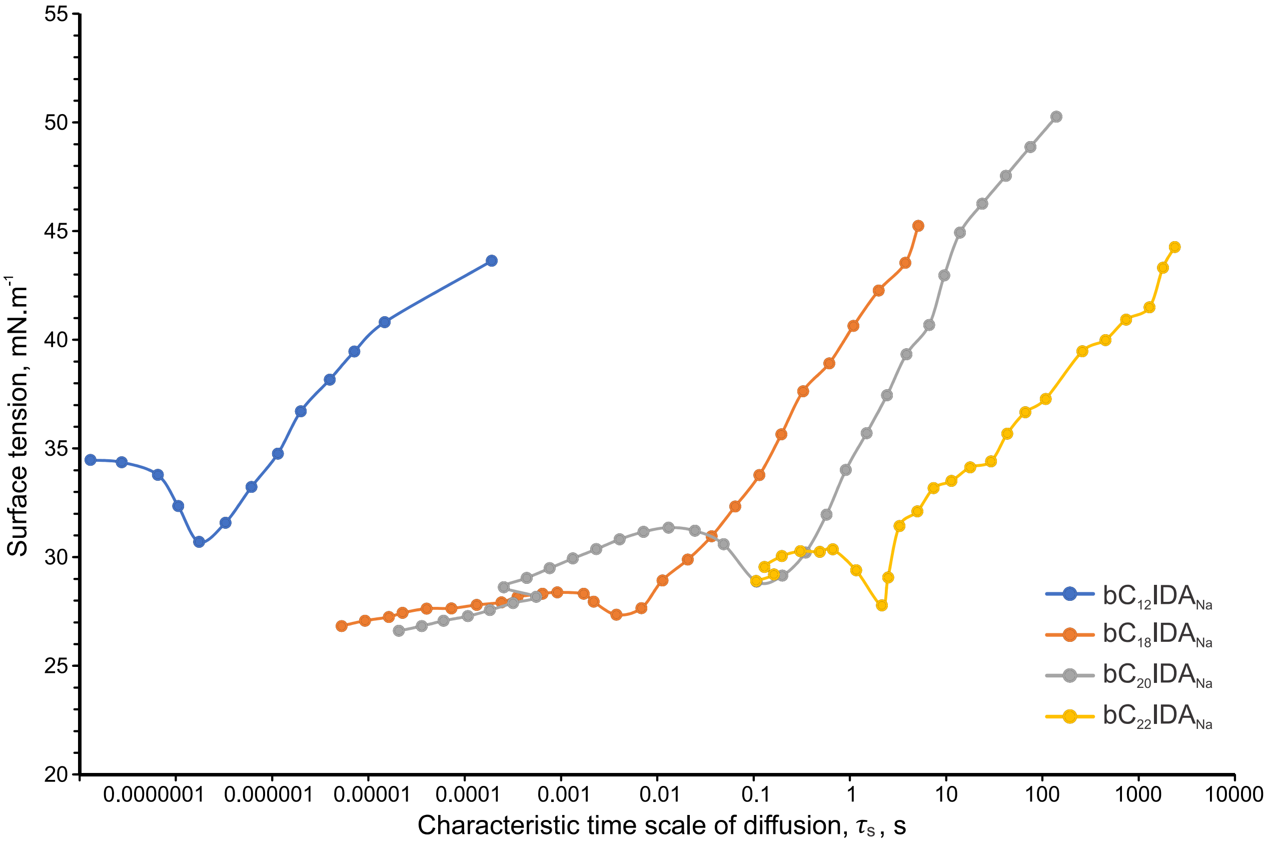

Figure S2.2. Characteristic timescale of diffusion curves of the Guerbet-type surfactants.

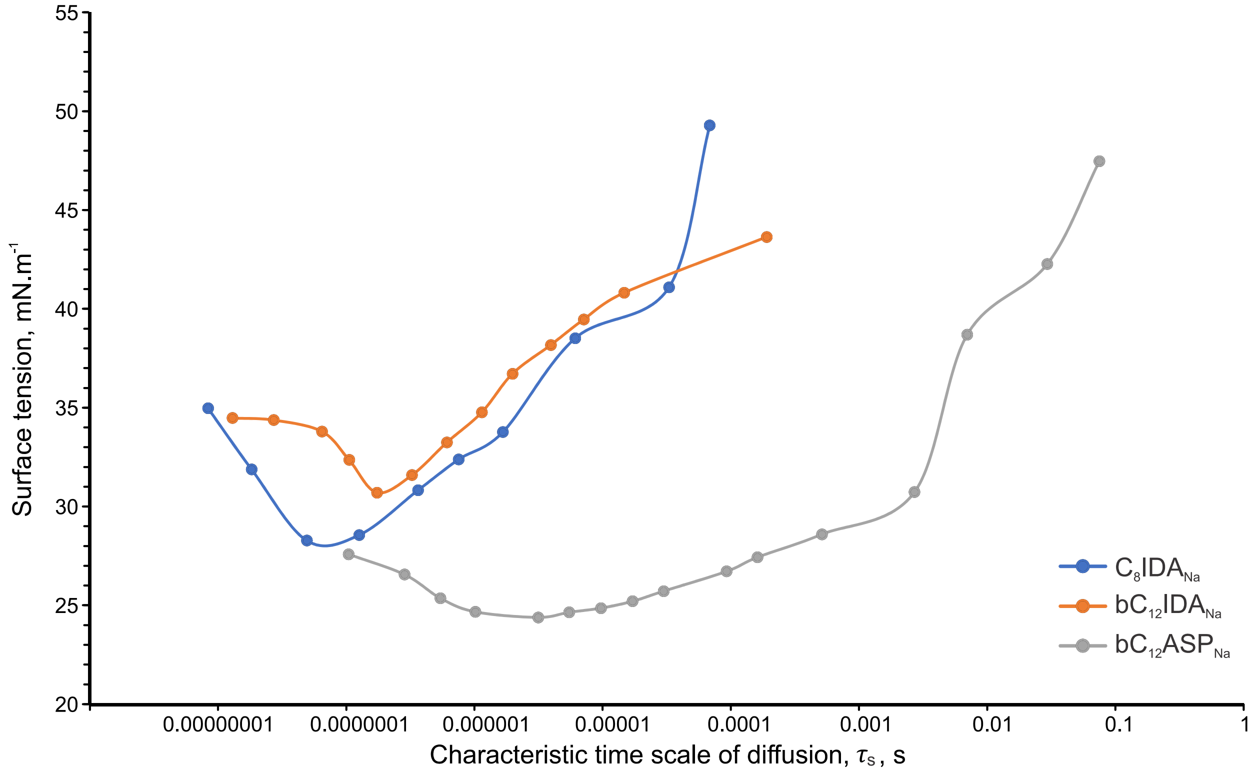

Figure S2.3. Characteristic timescale of diffusion curves of surfactants with an ECCL of 8.

Supporting Information 3: DOSY

Table S2. Method for DOSY-analysis.

Equipment	
NMR	Bruker Avance NEO 600 MHz
Probe	Direct observation
Settings	
Puls program	LEDbpgp2s
Temperature	25°C
TD	16384
NS	8–4096
d1	4–10 s
Δ (D20)	60 ms
δ (P30)	1 ms
D21	5 ms
Z_m	45 $G.cm^{-1}$
Increments	16 (5–95%)
Software	Topspin 4.4 and Dynamics Center (Bruker)

Table S3. CMCs determined via diffusometry and tensiometry.

Surfactant	CMC (Diffusometry)	CMC (Tensiometry)
C_8IDA_{Na}	230.5	207.8
$C_{13}IDA_{Na}$	7.5	9.5
$bC_{12}IDA_{Na}$	38.6	123.3
$bC_{18}IDA_{Na}$	1.1	1.0
$bC_{22}IDA_{Na}$	0.03	0.05
$bC_{12}ASP_{Na}$	37.7	42.9

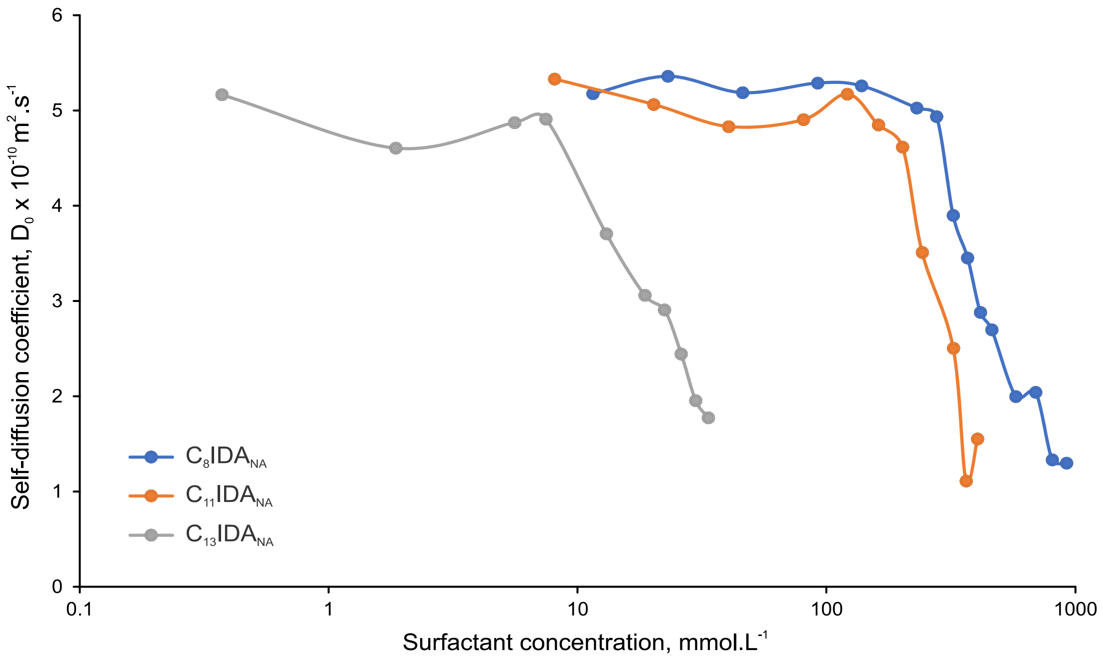

Figure S3.1. Self-diffusion concentration curves of the linear surfactants.

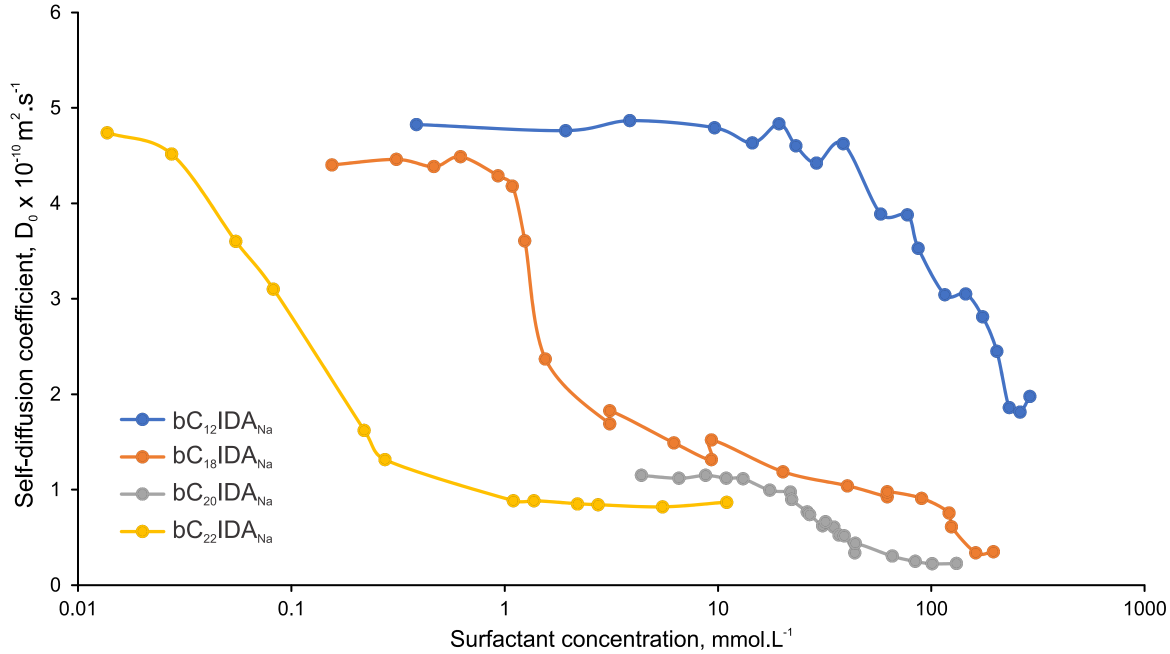

Figure S3.2. Self-diffusion concentration curves of the Guerbet-type surfactants.

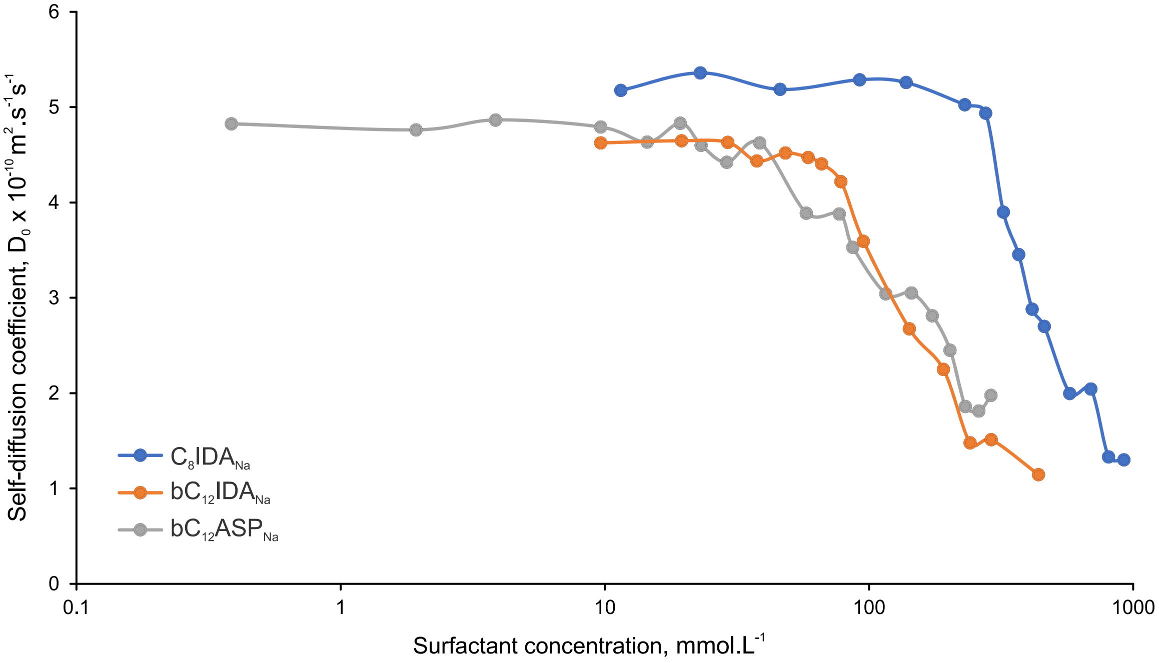

Figure S3.3. Self-diffusion concentration curves of surfactants with an ECCL of 8.

Supporting Information 4: Purity of surfactants

Table S4. Method for ^{1}H-NMR-analysis.

Equipment	
NMR	Bruker Avance NEO 600 MHz
Probe	Direct observation
**Settings**	
Puls program	LEDbpgp2s
Temperature	25 °C
TD	16384
NS	16–128
d1	4–10 s
Software	Topspin 4.4

Table S5. Method for LC-analysis.

Equipment		
LC	Agilent 1260 Infinity II	
Column	20 X 4.0 mm, 5 μm, 250 Å pore size, SUPELGUARD™	
**Settings**		
Mobile phase	Eluent A: H_2O 20 mM $NH_4HOOC/HOAc$; Eluent B: MeOH	
Flow	1.5 $ml.min^{-1}$	
Elution	Gradient	
Maximum pressure	800 bar	
Injection volume	2 μl	
Run time	20 min	
Column temperature	20 °C	
**Gradient elution settings**		
Time (min)	Eluent A (%)	Eluent B (%)
0–0.5	90	10
0.5–10	0	100
10–12	0	100
12–13	90	10
13–19	90	10

Table S6. Method for MS-analysis.

Equipment	
MS	Bruker microTOF-Q II
Settings	
Source type	ESI
Ion polarity	Negative
Scan range	50–1 600 *m*/z
End plate offset	-500 V
Collision cell RF	100.0 Vpp
Nebulizer	4.0 Bar
Dry heater	320 °C
Dry gas	N_2
Flow rate	12.0 $L.min^{-1}$

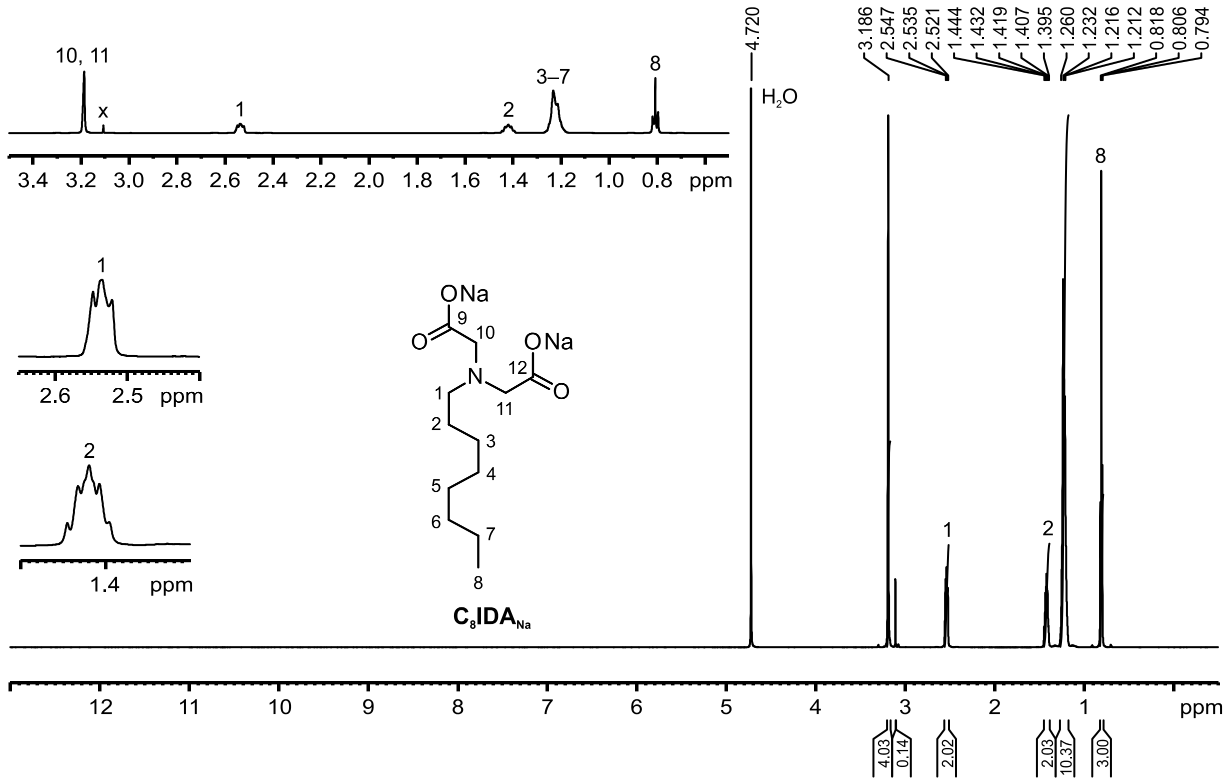

Figure S4.1. ^{1}H-NMR spectrum of C_8IDA_{Na} (600 mHz, D_2O).

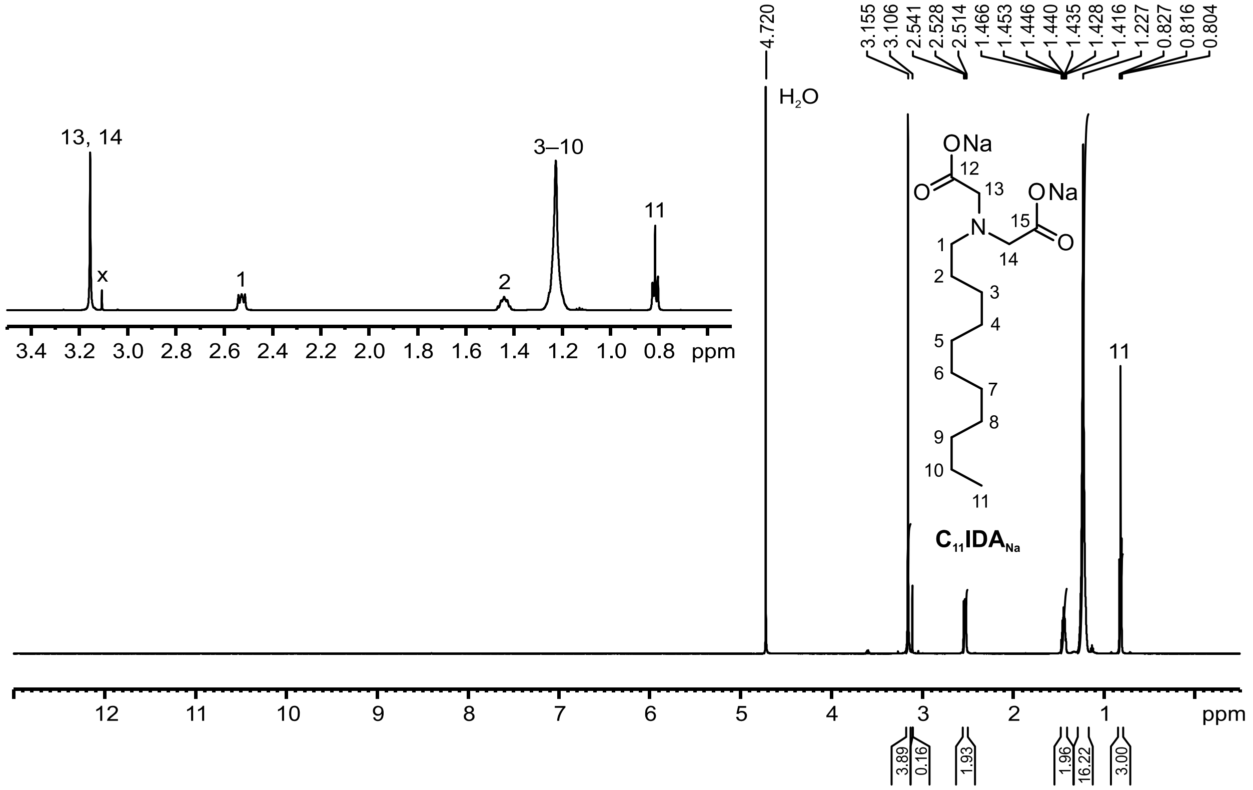

Figure S4.2. ^{1}H-NMR spectrum of $C_{11}IDA_{Na}$ (600 mHz, D_2O).

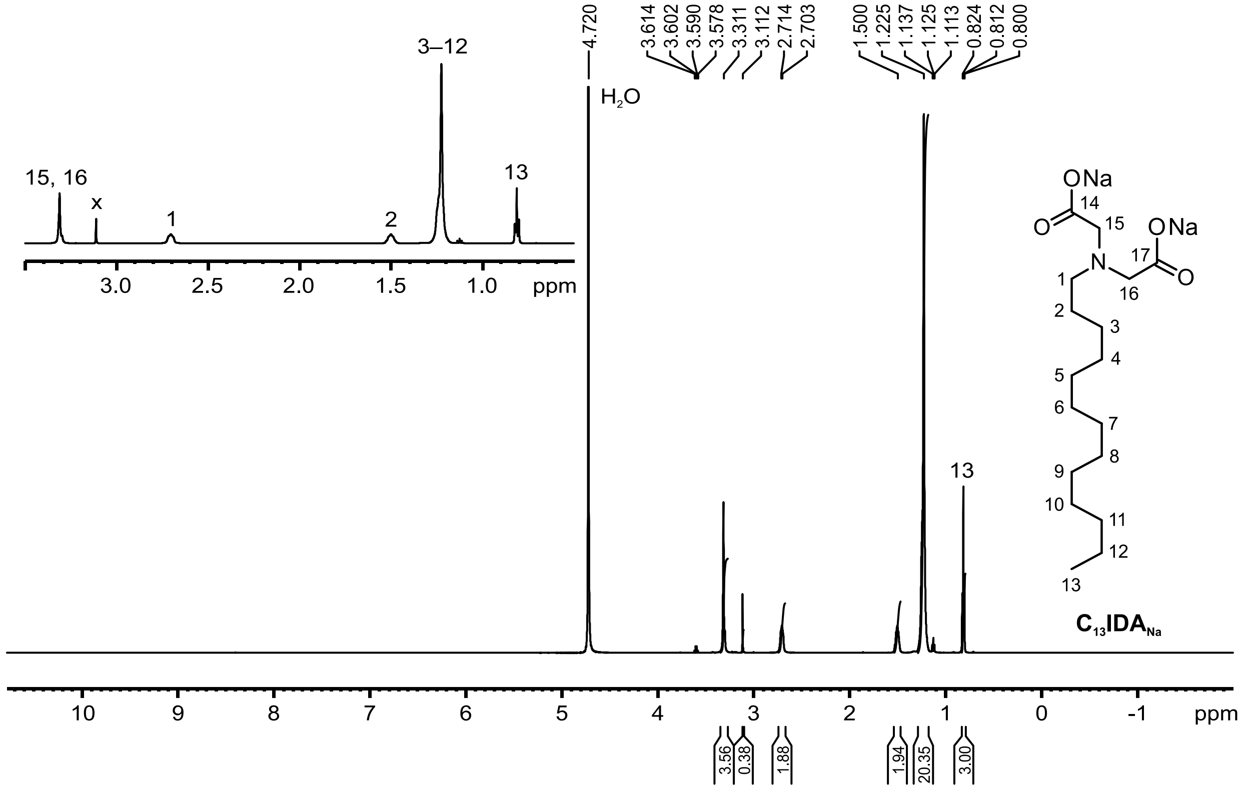

Figure S4.3. ^{1}H-NMR spectrum of $C_{13}IDA_{Na}$ (600 mHz, D_2O).

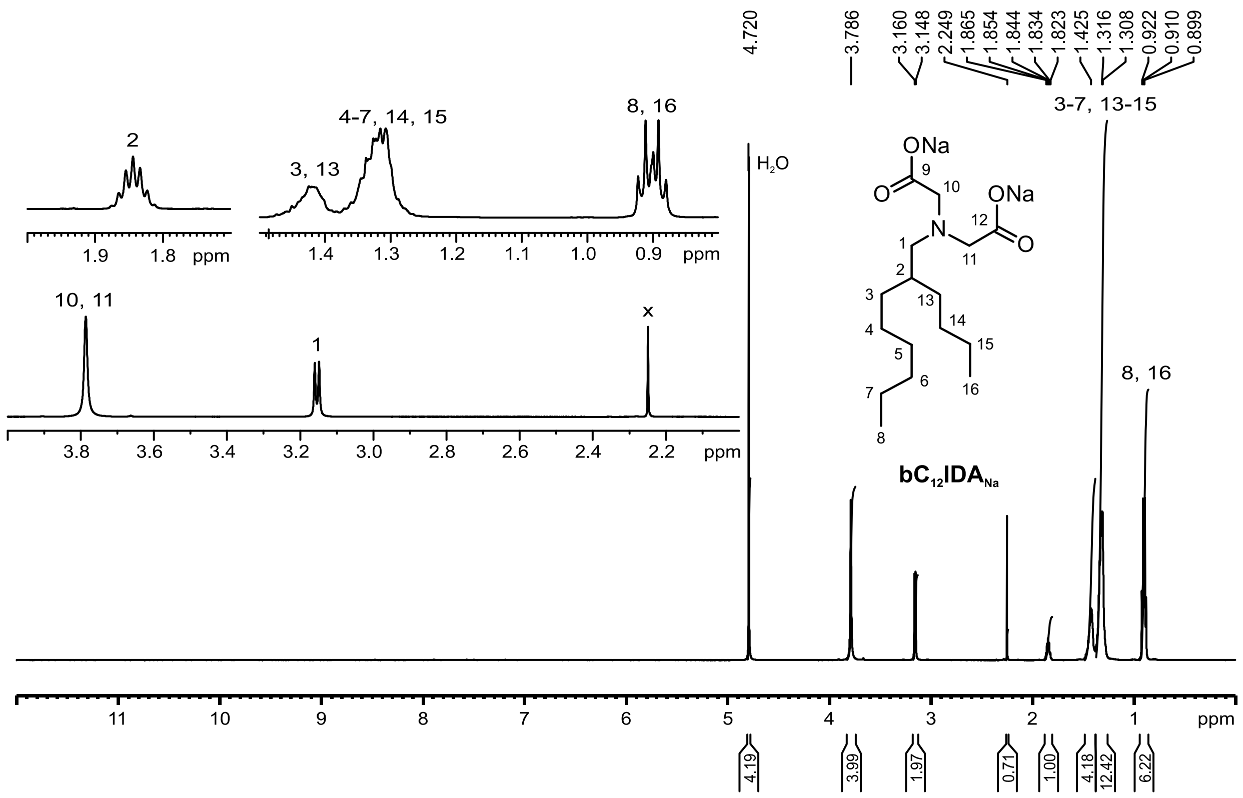

Figure S4.4. ^{1}H-NMR spectrum of $bC_{12}IDA_{Na}$ (600 mHz, D_2O).

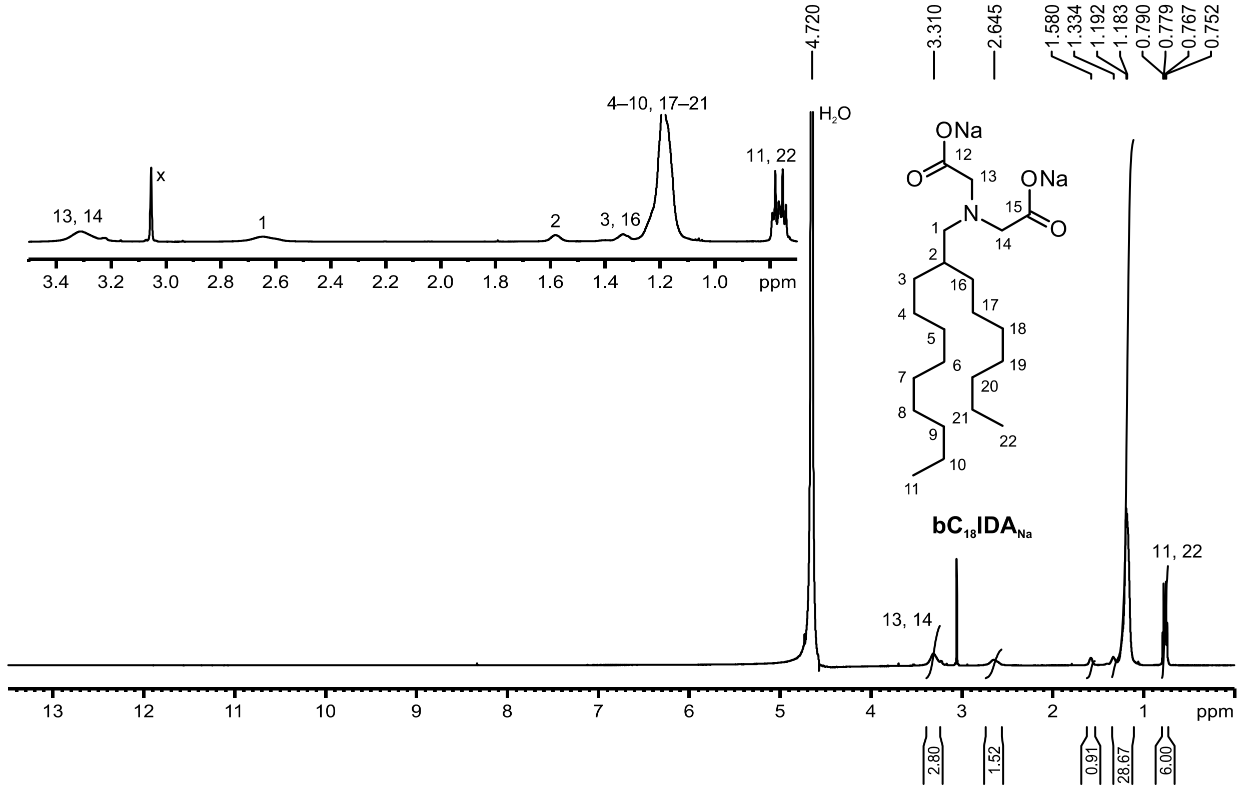

Figure S4.5. ¹H-NMR spectrum of $bC_{18}IDA_{Na}$ (600 mHz, D_2O).

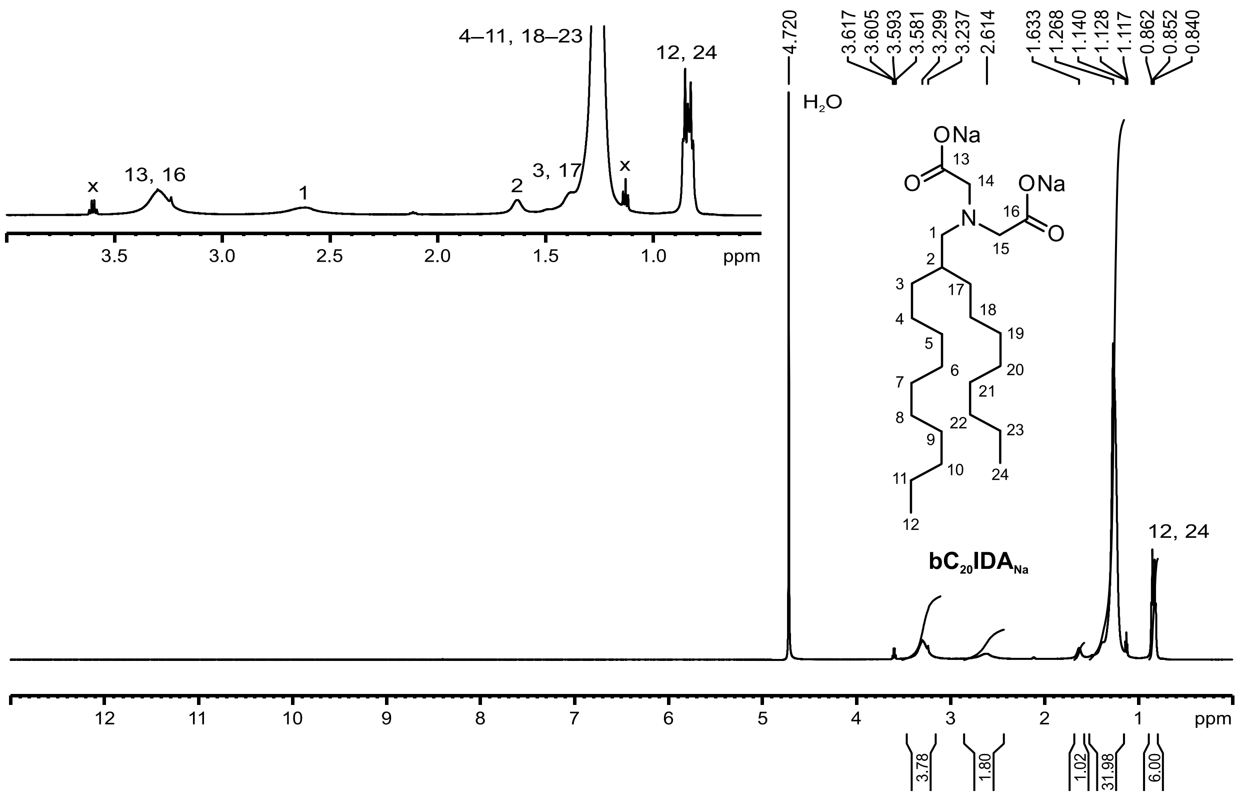

Figure S4.6. ¹H-NMR spectrum of $bC_{20}IDA_{Na}$ (600 mHz, D_2O).

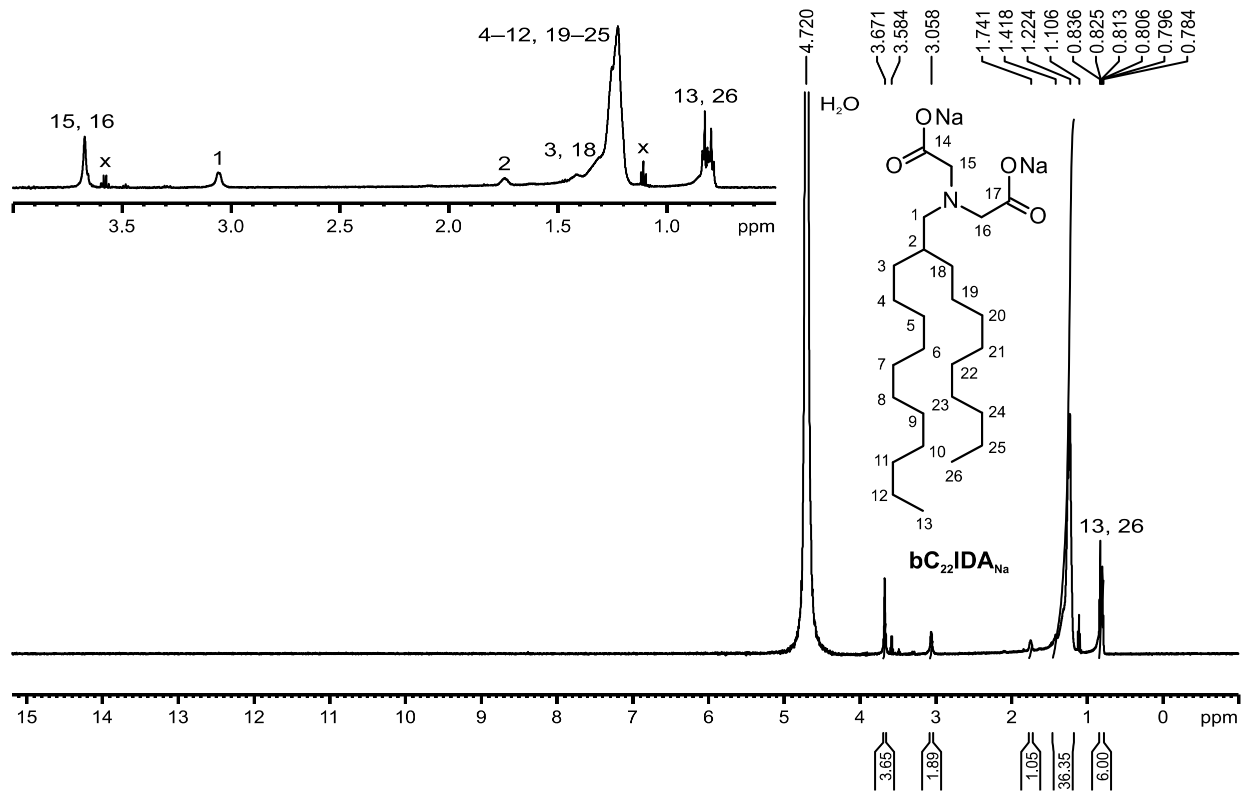

Figure S4.7. ^{1}H-NMR spectrum of $bC_{22}IDA_{Na}$ (600 mHz, D_2O).

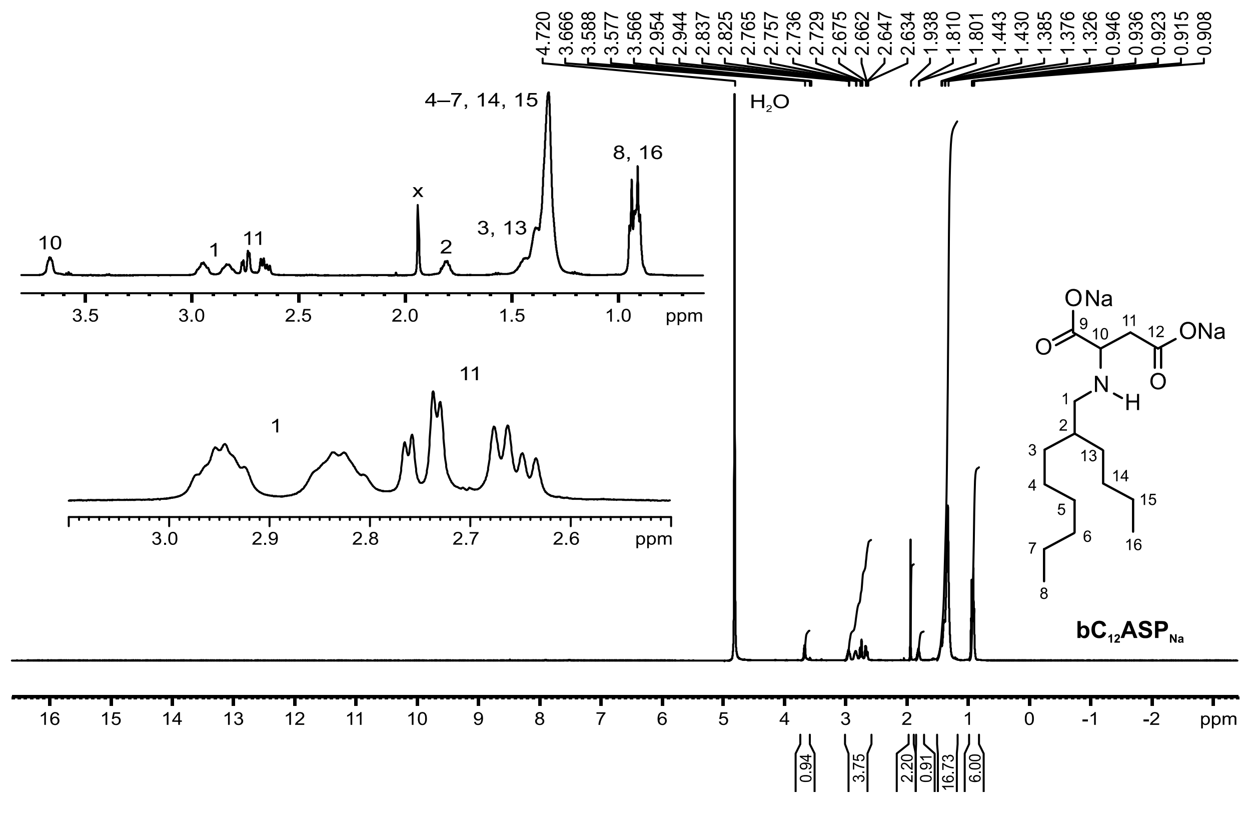

Figure S4.8. ^{1}H-NMR spectrum of $bC_{12}ASP_{Na}$ (600 mHz, D_2O).

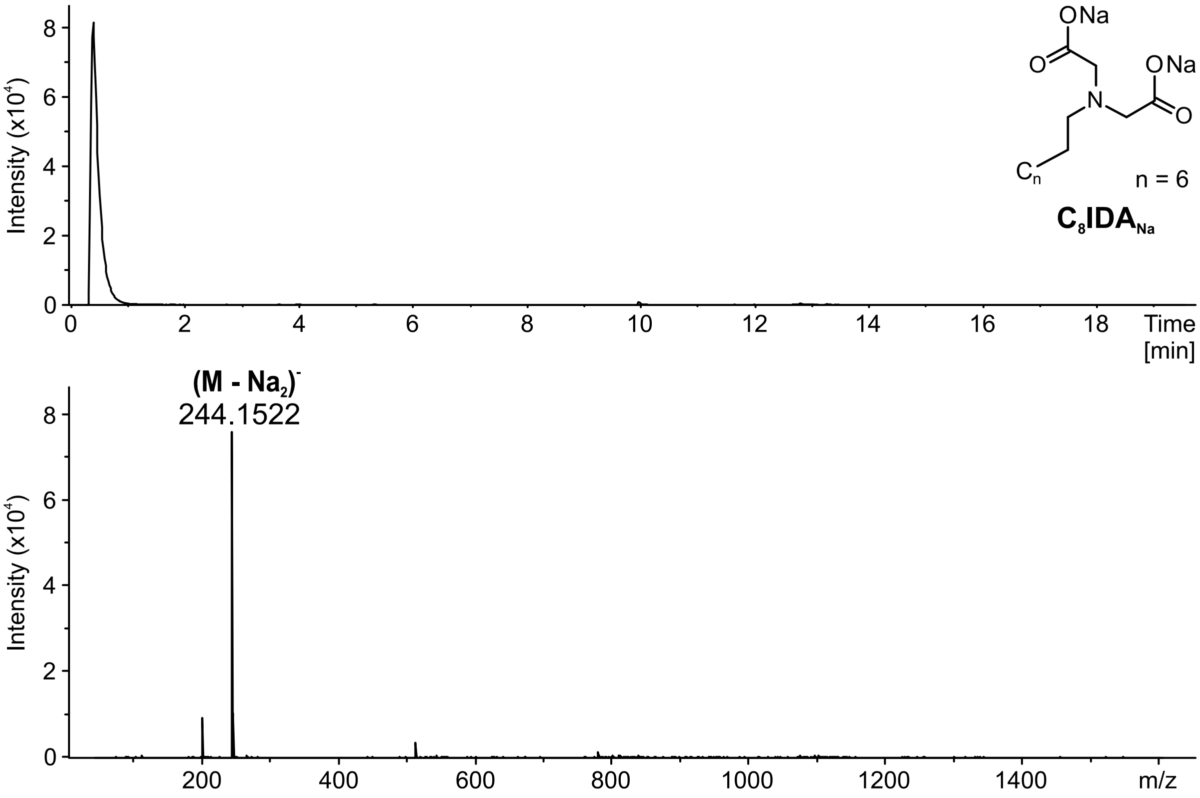

Figure S4.9. LC-MS spectra of C_8IDA_{Na}.

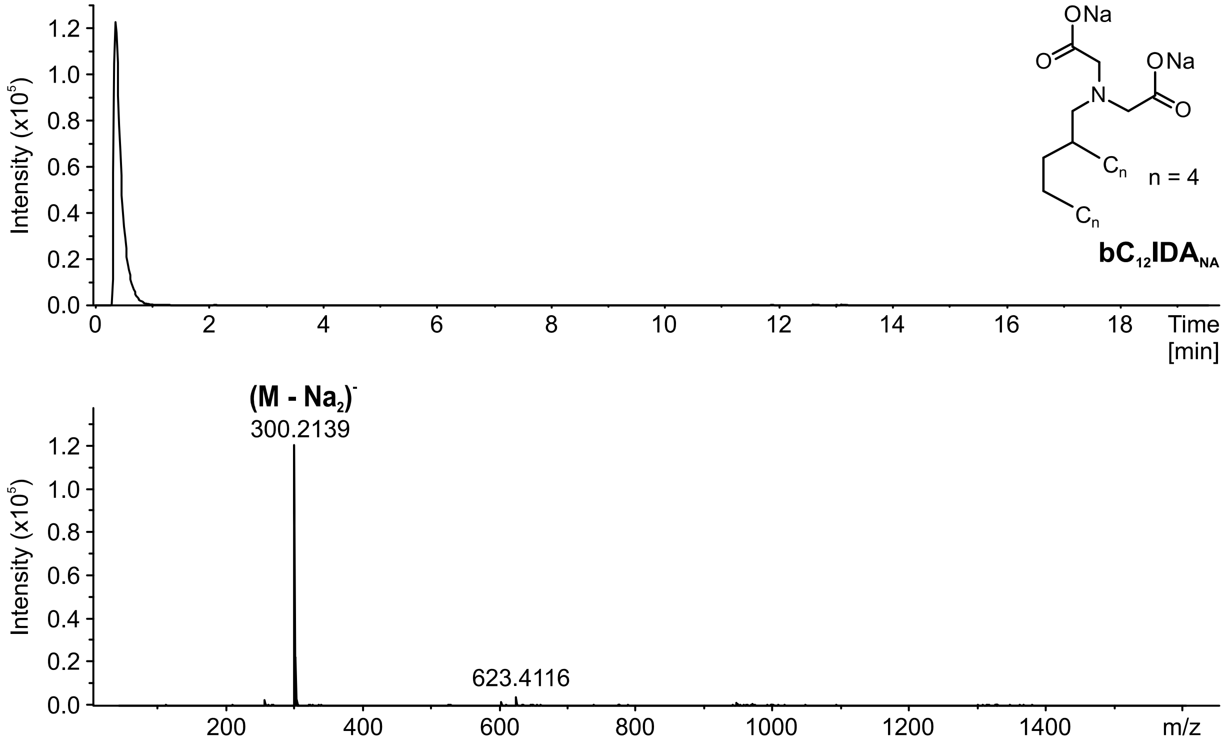

Figure S4.10. LC-MS spectra of $bC_{12}IDA_{Na}$.

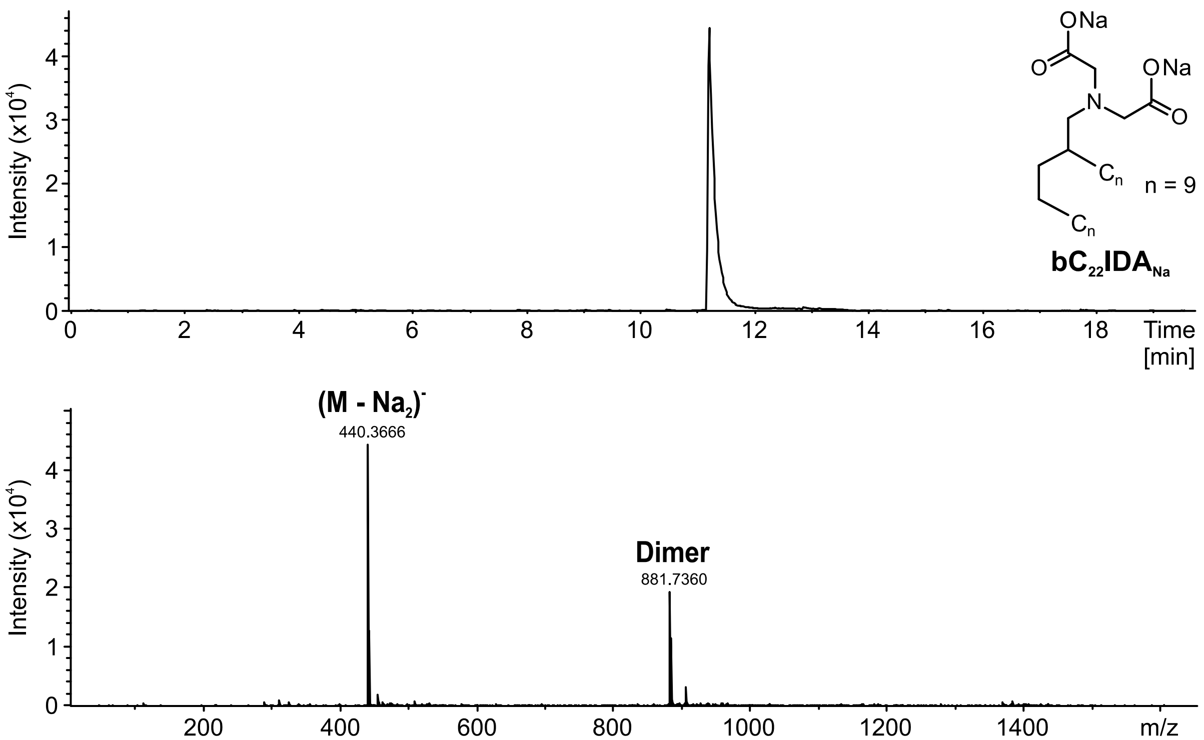

Figure S4.11. LC-MS spectra of $bC_{22}IDA_{Na}$.

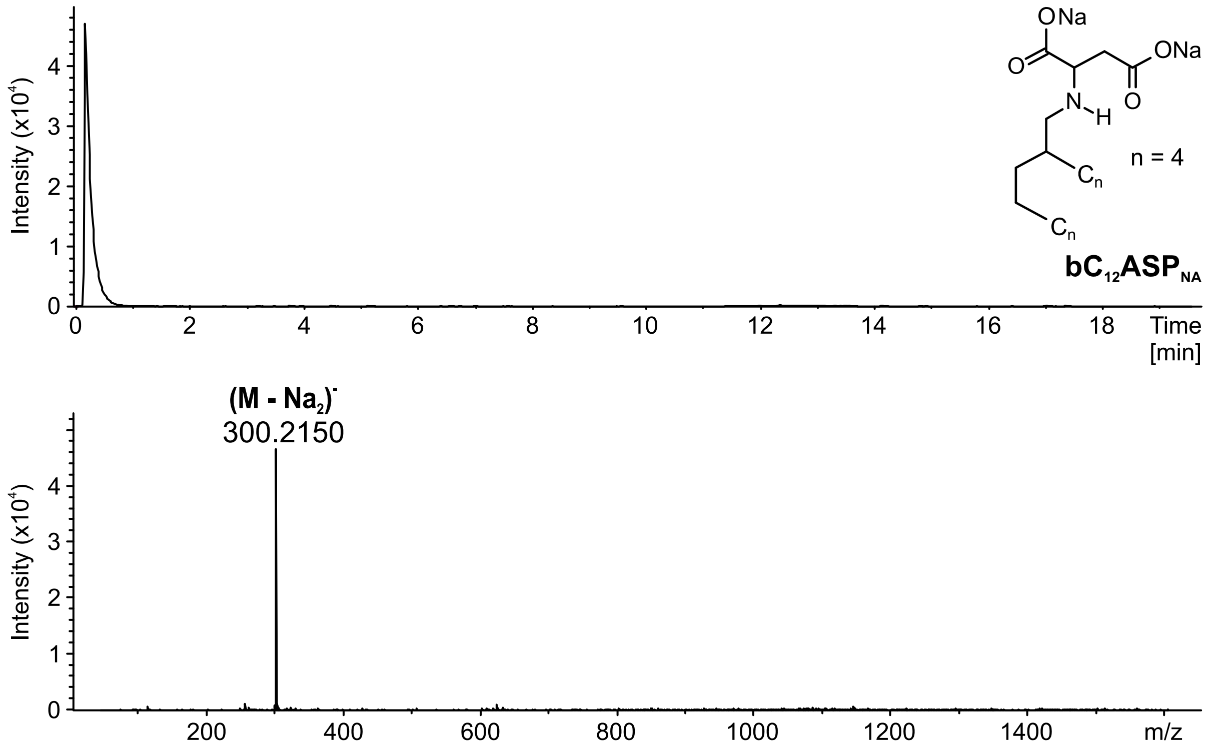

Figure S4.12. LC-MS spectra of $bC_{12}ASP_{Na}$.
